# Evolution of the SH3 Domain Specificity Landscape in Yeasts

**DOI:** 10.1371/journal.pone.0129229

**Published:** 2015-06-11

**Authors:** Erik Verschueren, Matthias Spiess, Areti Gkourtsa, Teja Avula, Christiane Landgraf, Victor Tapia Mancilla, Aline Huber, Rudolf Volkmer, Barbara Winsor, Luis Serrano, Frans Hochstenbach, Ben Distel

**Affiliations:** 1 EMBL/CRG Systems Biology Research Unit, Centre for Genomic Regulation-CRG, Barcelona, Spain; 2 Department of Molecular and Cellular Genetics, UMR7156, Université de Strasbourg and CNRS, Strasbourg, France; 3 Department of Medical Biochemistry, Academic Medical Center, University of Amsterdam, Amsterdam, The Netherlands; 4 Institut für Medizinische Immunologie, Humboldt-Universität zu Berlin, Berlin, Germany; University of South Florida College of Medicine, UNITED STATES

## Abstract

To explore the conservation of Src homology 3 (SH3) domain-mediated networks in evolution, we compared the specificity landscape of these domains among four yeast species, *Saccharomyces cerevisiae*, *Ashbya gossypii*, *Candida albicans*, and *Schizosaccharomyces pombe*, encompassing 400 million years of evolution. We first aligned and catalogued the families of SH3-containing proteins in these four species to determine the relationships between homologous domains. Then, we tagged and purified all soluble SH3 domains (82 in total) to perform a quantitative peptide assay (SPOT) for each SH3 domain. All SPOT readouts were hierarchically clustered and we observed that the organization of the SH3 specificity landscape in three distinct profile classes remains conserved across these four yeast species. We also produced a specificity profile for each SH3 domain from manually aligned top SPOT hits and compared the within-family binding motif consensus. This analysis revealed a striking example of binding motif divergence in a *C*. *albicans* Rvs167 paralog, which cannot be explained by overall SH3 sequence or interface residue divergence, and we validated this specificity change with a yeast two-hybrid (Y2H) assay. In addition, we show that position-weighted matrices (PWM) compiled from SPOT assays can be used for binding motif screening in potential binding partners and present cases where motifs are either conserved or lost among homologous SH3 interacting proteins. Finally, by comparing pairwise SH3 sequence identity to binding profile correlation we show that for ~75% of all analyzed families the SH3 specificity profile was remarkably conserved over a large evolutionary distance. Thus, a high sequence identity within an SH3 domain family predicts conserved binding specificity, whereas divergence in sequence identity often coincided with a change in binding specificity within this family. As such, our results are important for future studies aimed at unraveling complex specificity networks of peptide recognition domains in higher eukaryotes, including mammals.

## Introduction

Peptide recognition modules, like the Src homology 3 (SH3) domains, bind peptide motifs with low affinity and are predominantly found in signaling pathways, where they mediate transient protein-protein interactions that regulate cell proliferation and differentiation. These recognition domains often bind a core motif common to the domain family, surrounded by a number of specificity-determining residues that minimize cross-reactivity. SH3 domains generally bind to proline-rich sequences containing a core *PXXP* motif (where X is any amino acid) flanked by a positively charged residue [1,2]. Traditionally, these motifs have been further categorized as a Type I +*XXPXXP* motif (where + is a positively charged Arg or Lys) or a Type II *PXXPX*+ motif, distinguished by the location of the charged residue relative to the core motif [3,4]. Later, additional SH3 domain binding motifs were identified, including Type III domains, which bind to a polyproline motif without charged residues [5–8]. It thus became clear that the specificity landscape of SH3 domains is more diverse than previously appreciated. Recent studies, which capitalize on the continuous advances in high-throughput phage-display library development and sequencing technologies, generated up to 10 billion random peptides to explore the SH3 domain recognition landscape in an unbiased fashion and confirmed its complexity [9–11].

**Fig 1:**
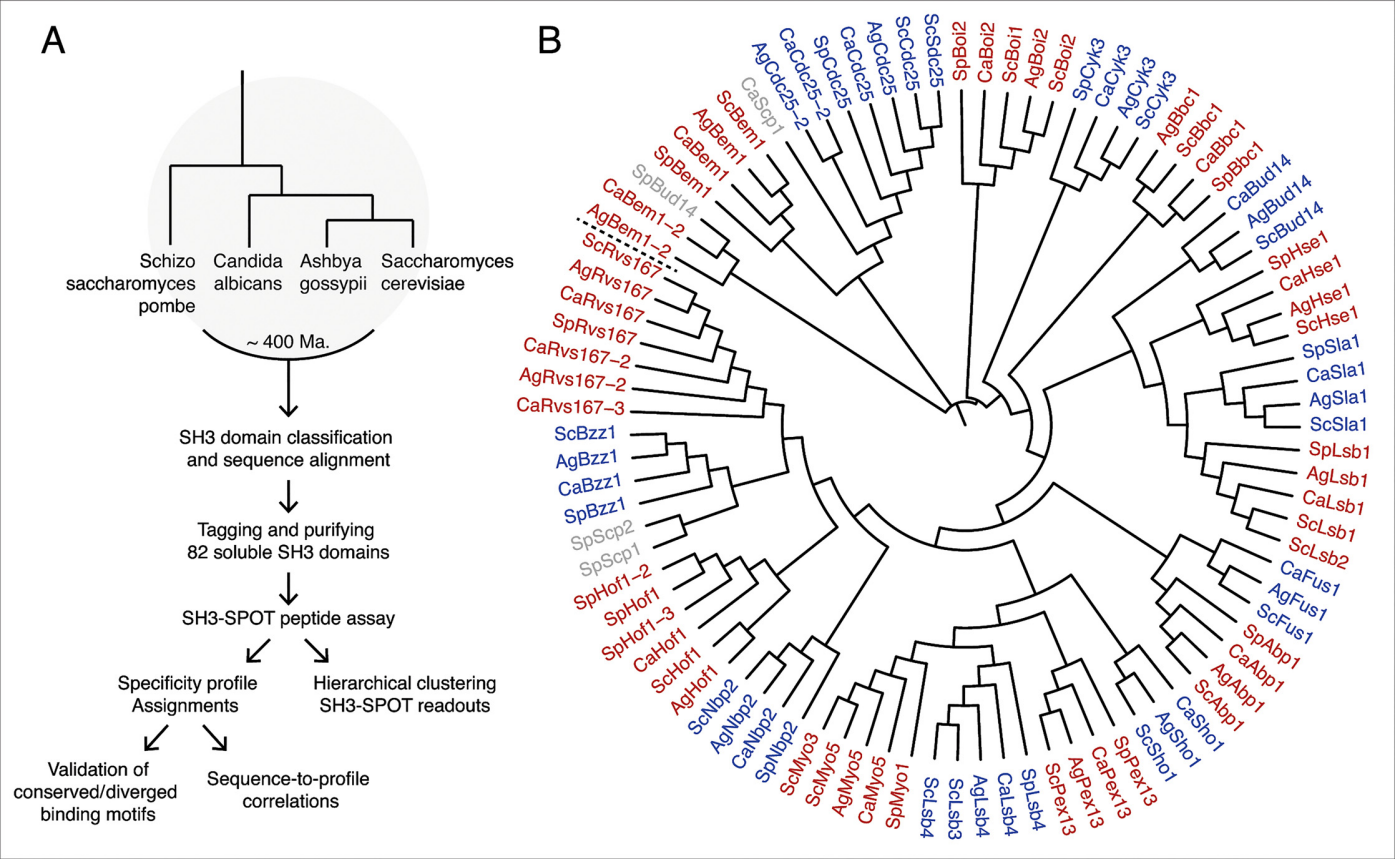
Approach used to characterize SH3 domain specificity conservation in four model yeasts. (**A**) Overview of the approach we used to characterize the SH3 domain specificity landscape in four yeast species that span an evolutionary distance of some 400 Ma. (**B**) Overview of all SH3 domain proteins in *S*. *cerevisiae* (*Sc*), *A*. *gossypii* (*Ag*), *C*. *albicans* (*Ca*) and *S*. *pombe* (*Sp*). The dendrogram derived from their full multiple sequence alignment illustrates the diverging sequence conservation of orthologs and paralogs, analogous to the evolutionary distance among the four different yeasts. Alternating colors of red and blue indicate conserved families. Previously non-described SH3 domain containing proteins (Scp) that could not be confidently assigned to a family are shown in grey.

To explore how SH3 domain specificity landscapes evolve, we compared SH3 binding specificity in four model yeast species that have had their entire genome sequenced and mapped: *Saccharomyces cerevisiae* [12], *Ashbya gossypii* [13], *Candida albicans* [14], and *Schizosaccharomyces pombe* [15] (Fig 1A). *S*. *cerevisiae* is the best studied unicellular eukaryote and it divides exclusively by budding, hence the name budding yeast. *A*. *gossypii* is a filamentous plant pathogen belonging to the *Saccharomycetes* that predates the whole-genome duplication, and is evolutionarily closely related to budding yeast. *C*. *albicans* is a human fungal pathogen that can switch between bud-like and hyphal growth. *S*. *pombe* is the second best studied yeast and evolutionarily most distant from the other yeasts. It shows bipolar growth and divides by fission, hence the name fission yeast. Together, these yeasts encompass roughly 400 million years of evolution [16].

We first identified all SH3 domains in the aforementioned four yeasts (109 domains), constructed multiple structurally informed sequence alignments, and compared the conservation of documented binding motifs in SH3 sequences [17]. Then, we defined SH3 domain borders for GST-tagged expression constructs and purified all soluble SH3 domains to perform quantitative peptide binding assays (SH3-SPOT). The quantitative SPOT results for a total of 82 SH3 domains allowed us to compute a pair-wise correlation matrix, which was hierarchically clustered to assess the conservation of general specificity classes across these four species and binding profile similarities within a single family. In addition, the SH3-SPOT assay data allowed us to construct position-weighted matrices (PWMs) from top hits, visualized as motif logos, to more accurately compare specificity conservation within a single family as well as estimate binding motif conservation in homologous binding partners of SH3 proteins. To validate the results of the SPOT assay we performed an actin polymerization assay and yeast two-hybrid (Y2H) assay for the Myo5 and Rvs167 families, respectively, and showed excellent agreement between these three experiments. Finally, by comparing pairwise SH3 domain sequence similarity and binding profile correlation within a single family, we aimed to gain insight into how the intricate relationship between these features has evolved in the context of conserved SH3 protein homologs in yeasts.

## Results and Discussion

### Conservation and duplication of SH3 domains

We identified all SH3 domain proteins in the four yeast species (See Materials and Methods) and investigated whether their overall predicted domain architecture and sequence identity are conserved (Fig 1B). We found that *S*. *cerevisiae* has more duplicated genes (Cdc25/Sdc25, Boi2/Boi1, Lsb4/Lsb3, Lsb1/Pin3, Myo5/Myo3) than the other three species, which is consistent with the whole-genome duplication event in the *S*. *cerevisiae* branch [18,19]. *S*. *pombe*, which has the smallest genome (4,824 protein-coding genes) of these four yeasts [15,20], lacks two homologs (Sho1, Fus1) present in the other three species. Besides these two exceptions, we found that all *S*. *cerevisiae* SH3 proteins could be mapped to orthologs in, *A*. *gossypii*, *C*. *albicans*, and *S*. *pombe* (S1 Table). To facilitate the cross-species comparison in this study, from here on we will refer to homologs in *A*. *gossypii* (*Ag*), *C*. *albicans* (*Ca*), and *S*. *pombe* (*Sp*) with names according to their *S*. *cerevisiae* (*Sc*) based family names.

With respect to gene duplications, we observed that *A*. *gossypii* and *C*. *albicans* each have an additional Bem1 paralog (*Ag*Bem1-2 and *Ca*Bem1-2) and a Cdc25 paralog (*Ag*Cdc25-2 and *Ca*Cdc25-2). Furthermore, the Hof1 family has three paralogs in *S*. *pombe* (*Sp*Hof1, *Sp*Hof1-2, and *Sp*Hof1-3), of which the former two have been studied extensively. Their SH3 domains are collectively essential and functionally interchangeable in *S*. *pombe* cytokinesis, recognizing selected Type I interactors with the motif +*XLPXXP* [21]. The Rvs167 family is most represented with two additional Rvs167 paralogs (*Ca*Rvs167-2 and *Ca*Rvs167-3) in *C*. *albicans* and one additional paralog (*Ag*Rvs167-2) encoded by the *A*. *gossypii* genome. Unfortunately, we were unable to determine whether these duplicated genes originate from a common yeast ancestor and were lost in *S*. *pombe* and *S*. *cerevisiae*, or whether the gene duplication occurred independently in *C*. *albicans* and *A*. *gossypii*. Interestingly, *Ca*Rvs167-3 is observed in a number of yeast species closely related to *C*. *albicans*, which indicates that the duplication occurred after a speciation event of a common *C*. *albicans* ancestor. In a recent study, Wapinski *et al*. [19] point out that duplicated genes rarely diverge with respect to biochemical function (neo-functionalization) but more commonly specialize in a partial function of the ancestral gene (sub-functionalization). Indeed, cells harboring a deletion of *CaRVS167-2* or *CaRVS167-3* do not show a phenotype [22,23], whereas cells with a deletion of *CaRVS167* are deficient in endocytosis [24]. Future experiments are needed to explore the precise roles of the individual Rvs167 paralogs in *A*. *gossypii* and *C*. *albicans*. Rvs167 proteins have besides an SH3 domain an N-terminal BAR (Bin, Amphiphysin, Rvs) domain (PFAM 03114; SMART 00721), enabling them to form homodimers or heterodimers [25,26], and to interact with cellular membranes [27,28]. We found that the *C*. *albicans* genome also contains about three times more BAR-domain containing proteins (S1 Fig). BAR proteins play an essential role in membrane curvature formation during clathrin-mediated endocytosis [29]. The duplication of genes encoding proteins with a BAR-SH3 domain architecture suggests a more tightly regulated and complex system of endocytosis in *C*. *albicans*.

**Fig 2:**
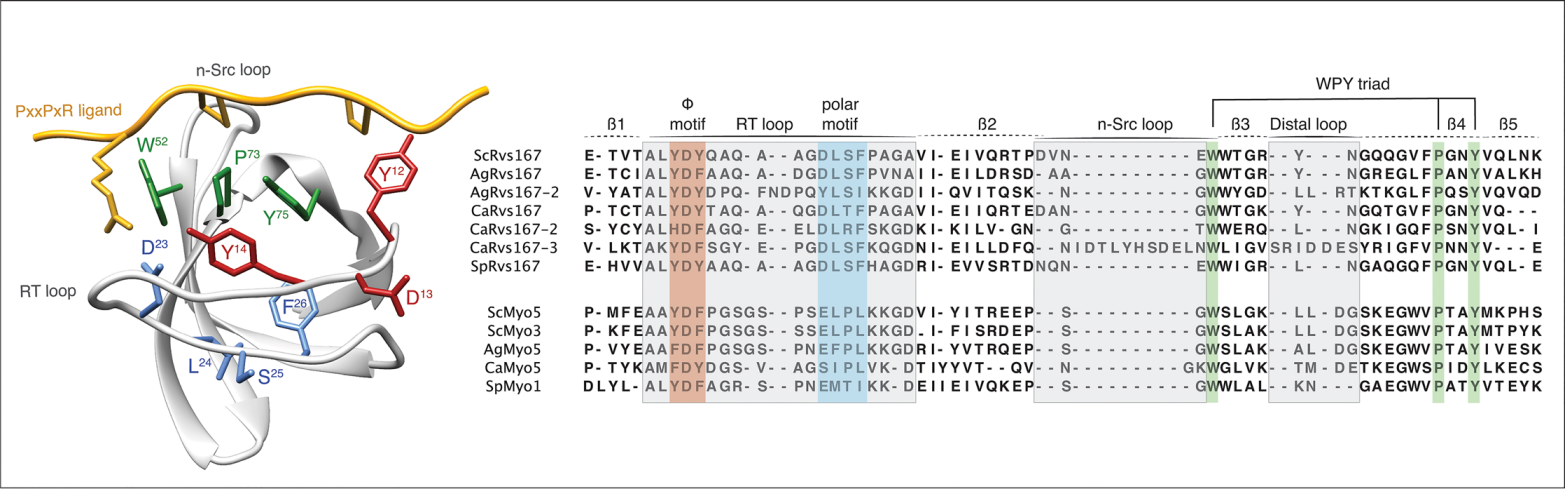
Structure-based alignments of SH3 domains and binding site annotations. A structural model of the *Sc*Lsb3 SH3 domain (left) (PDB: 1SSH) with its PxxPxR ligand (yellow) shows the three canonical SH3 domain binding site motifs: the WPY triad (green) and the hydrophobic (red) and polar motifs (blue) of the RT loop. Structure-based sequence alignments of the highly conserved Rvs167 and Myo5 families, annotated with the three canonical binding motifs and the three loop locations (grey), reveal an unusually large insertion in the n-Src loop of *Ca*Rvs167-3 (right).

Divergence of a specificity-determining sequence motif between homologous domains often provides strong evidence for altered peptide-ligand recognition. Therefore, we constructed multiple sequence alignments for each SH3 domain family (see Materials and Methods) as an aid to annotate the three main ligand-binding motifs that shape the SH3 binding pocket and determine specificity (Figs 2 and S2). We identified the presence of the highly conserved WPY triad that forms a groove binding one of the canonical prolines, and two motifs in the RT loop: the aromatic motif that usually forms a second proline binding-groove, and the polar motif, a major specificity-determining factor [30]. We also annotated the loop lengths of the RT and n-Src loops, based on homology models, as they are known to be determinants for SH3 specificity as well. Not surprisingly, the majority of conserved SH3 domains also show highly conserved ligand-binding motifs among homologs in the four species.

### SPOT analysis

**Fig 3:**
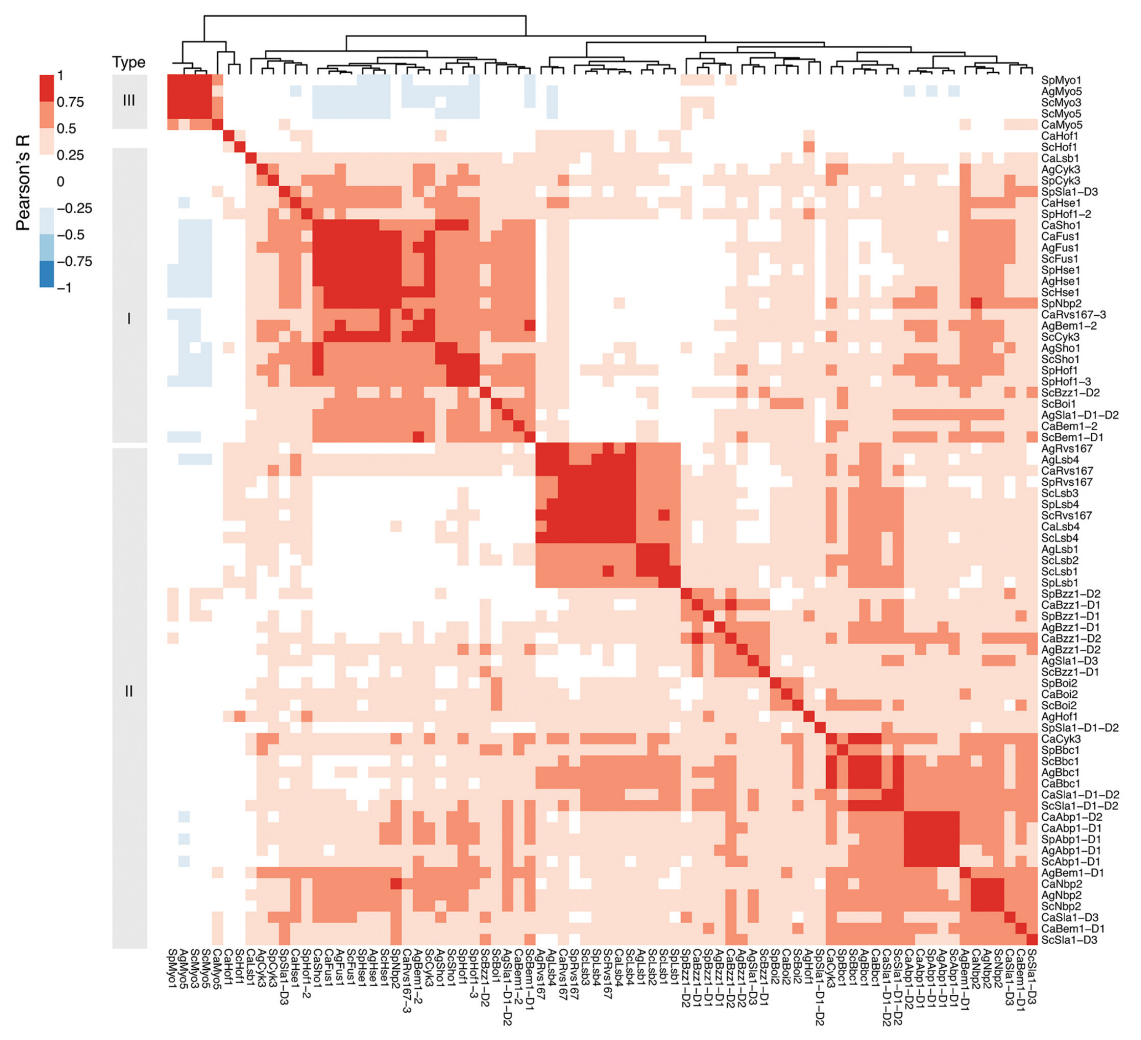
Clustering of SH3 SPOT binding profiles reveals conservation of the canonical specificity classes. A clustered heat map of normalized SH3 SPOT binding profile correlations across the four yeast species shows three distinct clusters corresponding to the three canonical SH3 specificity classes: Type I (+xxPxxP), Type II (PxxPx+), and Type III (polyproline), and a generally tight correlation between SH3 domains of the same family.

**Fig 4:**
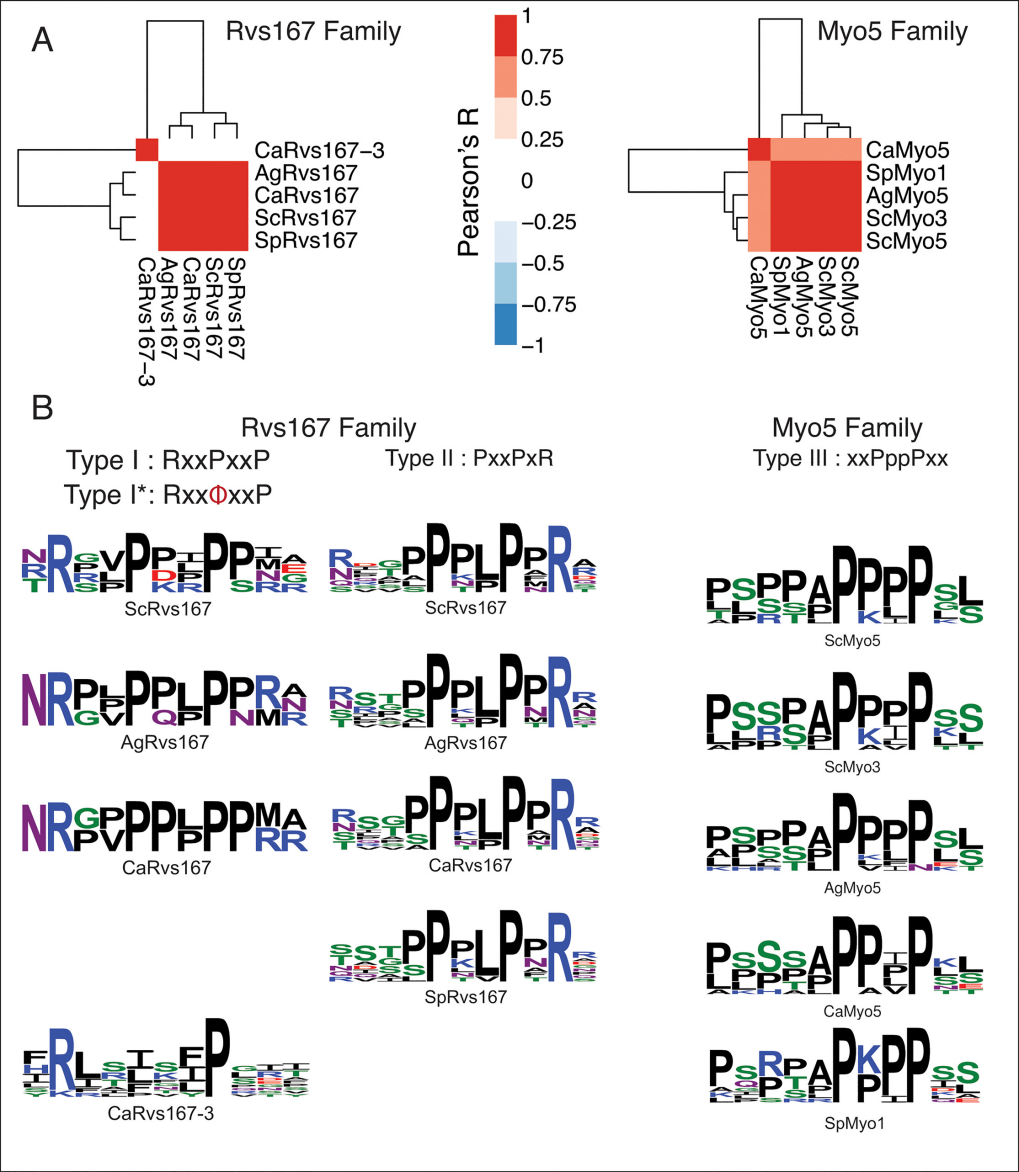
Within-family comparisons of specificity profiles highlight a novel diverged specificity class for *Ca*Rvs167-3. (**A**) Separately clustered heat maps of the Rvs167 and Myo5 families show that both families have a high degree of binding profile conservation among orthologs, with the exception of *Ca*Rvs167-3, whose binding profile does not correlate with any of the Rvs167 orthologs. (**B**) Specificity logos built from manual alignments of the top 10 binding peptides show that, with the exception of *Sp*Rv167, all Rvs167 binding peptides could be aligned as Type I and II profiles (left). The *Ca*Rvs167-3 binding profile forms a distinct Type I-like (Type I*) class, characterized by the presence of a hydrophobic residue instead of the first proline. All Myo5 ortholog binding profiles show a clear disposition for a poly-proline motif, devoid of charged residues (right).

Next, we performed SPOT peptide assays with all soluble SH3 domain constructs (see Materials and Methods) to compare the binding specificities for homologous domains across the four species. To probe SH3 binding-specificity in yeast we used an established library of 292 SH3 binding 15-mers, which were previously mined from the *S*. *cerevisiae* proteome and tested for SH3 binding [9]. Of the 109 predicted SH3 domains, 89 domains could be purified in sufficient amounts for SPOT analysis. We obtained data for 82 domains, resulting in an overall coverage of ~75% of all SH3 domains across the four species (S2 and S3 Tables; Figures A and B in S1 File). To accurately compare the results of all SPOT assays for all SH3 domains, we normalized the dataset in batch by median-scaling the distributions of log-transformed SPOT intensities, averaged over biological replicates. Then, we computed a pair-wise Pearson correlation matrix among the SPOT readouts of SH3 domains that were represented in at least 3 out of 4 species within a family (74 out of 82) and clustered this matrix with a hierarchical clustering algorithm (see Materials and Methods). The results of the clustered correlation coefficients were represented in a heat map (Fig 3). We observed that the main clusters on this heat map strikingly represent the three major SH3 domain specificity classes: Types I, II, and III (poly-proline). Based on this classification scheme we compared our specificity type assignments to those recently published for *S*. *cerevisiae* [9]. Overall we found that the specificity type assignments were similar, with the exception of those for *Sc*Fus1 and *Sc*Hse1, which may be due to the use of a slightly different library of SH3-SPOT peptides. Surprisingly, many domain families clustered very tightly within these broad classes, which suggest that specificity niches, optimized to minimize cross-reactivity within species, are often conserved over large evolutionary distances. In our analysis, specificity profiles for most SH3 domain families are well conserved (Abp1, Bbc1, Boi2, Cyk3, Fus1, Hse1, Lsb1, Lsb4, Myo5, Nbp2, Rvs167, and Sho1) while weakened profile conservation seems to be the exception (Bem1, Bzz1, Hof1, and Sla1) (Figure C in S1 File). Interestingly, the unusual polyproline-binding signature for the *S*. *cerevisiae* myosin SH3 family is highly conserved and also occupies a unique place in the SH3 specificity landscape of *A*. *gossypii*, *C*. *albicans* and *S*. *pombe*. However, we also observed a number of striking anomalies from the globally clustered profile. For instance, the SH3 domain of *Ag*Bem1-2 accumulates major changes in the main binding pocket without an apparent change in ligand binding specificity. Another striking example is *Ca*Rvs167-3. The *C*. *albicans* paralog of the highly conserved Rvs167 family clearly clusters alongside all Type I motifs (Fig 3). To examine this in more detail, we selected for all Rvs167 domains the top 10 ligands, based on their intensity values, and aligned them by hand (Figs 4 and S3). In agreement with previously published studies [9,31] the top binding peptides of all Rvs167 domains could be aligned as a Type I or Type II motif except for *Sp*Rvs167 and *Ca*Rvs167-3. In contrast to most Rvs167 family members, which display a dominant Type II motif supported by a secondary Type I motif, *Ca*Rvs167-3 adopts a dominant Type I-like motif only (Fig 4B). We call this motif Type I-like because, despite the lack of the first proline, we observe a clear preference for a positively charged residue in the expected position of a Type I motif. Given that the SH3 domain sequences of *Ca*Rvs167-3 and *Ca*Rvs167 are quite similar, except for the presence of a large insertion in the n-Src loop of *Ca*Rvs167-3, we hypothesize that the change in ligand recognition is caused by this loop insertion (Fig 2). Unfortunately, we were unable to expand on this argument in the absence of a three-dimensional structure or a reliable model of the *Ca*Rsv167-3 SH3 domain bound to a Type I-like ligand.

### Myo5 and Rvs167 binding validation assays

#### Ex vivo actin polymerization study for myosins

**Fig 5:**
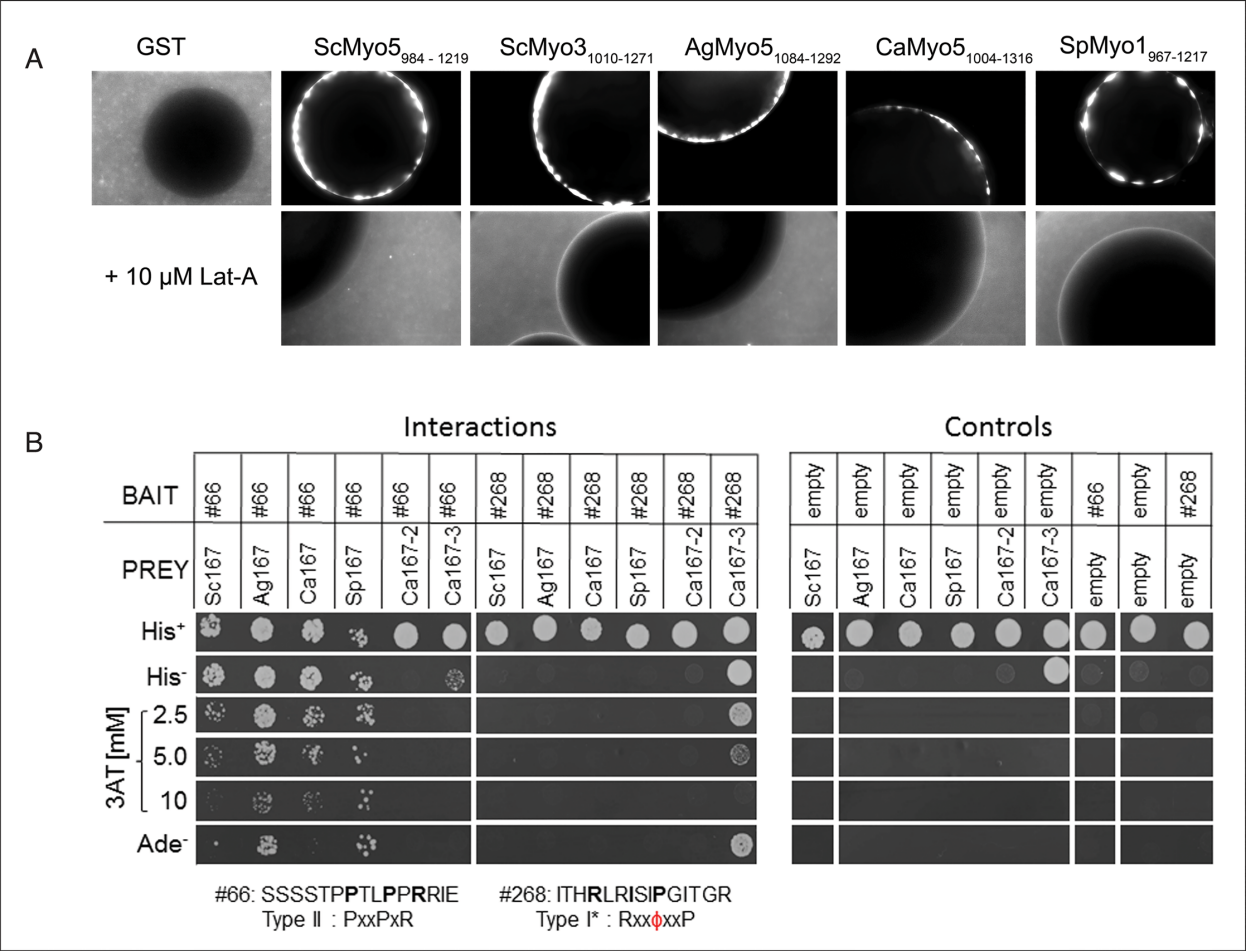
Myo5 and Rvs167 binding validation. (**A**) Sepharose-bead bound GST or GST-tagged C-terminal myosin type I tails of *Sc*Myo5 (984–1219), *Sc*Myo3 (1010–1271), *Ag*Myo5 (1084–1292), *Ca*Myo5 (1004–1316) and *Sp*Myo1 (1967–1217) were incubated with a total protein extract of *S*. *cerevisiae* supplemented with TRITC-labeled actin. The fluorescent halos around the beads (sized 50–150 μm) show the ability of the myosin type I tails of the four different yeast species to recruit active actin polymerization machinery to the beads while the negative control GST does not. Addition of 10 μM Latrunculin A inhibits actin polymerization. (**B**) Yeast two-hybrid strains co-transformed with the indicated bait and prey constructs were spotted (~10^4^ cells) on minimal plates with histidine (His^+^), without histidine (His^−^), without histidine containing 2.5, 5, or 10 mM 3-amino-1,2,4-triazole (3AT), or without adenine (Ade^–^). Weak interactors activate only the *HIS3* reporter and show growth on His^−^plates, while strong interactors activate both *HIS3* and *ADE2* reporters and show growth on His^−^plates containing 3AT or on Ade^−^plates. Note that *Ca*Rvs167-3 SH3 shows weak self-activation as revealed by growth on His^−^plates in the presence of an empty bait plasmid.

To experimentally confirm the conservation of the binding specificity of the type I myosin we chose an *ex vivo* approach established by Geli and colleagues [32]. This method assesses the ability of sepharose-bound proteins to induce actin polymerization using fluorescently labeled actin. We demonstrate that the SH3-containing C-terminal Myo5 tails of all four species were able to induce actin polymerization when incubated with total *S*. *cerevisiae* protein extract as revealed by a fluorescence halo formation around the sepharose beads (Fig 5A). As the interaction of the Myo5 SH3 domain with the Wiskott-Aldrich syndrome protein [WASP]-interacting protein (WIP) homolog Vrp1 was shown to be essential for the initiation of actin polymerization in *S*. *cerevisiae* [32], these data validate an interaction between *Sc*Vrp1 and all four Myo5 SH3 domains, confirming an interspecies conservation of the binding specificity of the type I myosins.

#### Motif validation for Rvs167 by yeast two-hybrid

To obtain independent experimental validation for the Rvs167 binding specificity we performed a yeast two-hybrid assay with peptides that showed high intensity values in the SPOT analysis (Fig 5B). We selected the Type II peptide #66 (SSSSTP**P**TL**P**P**R**RIE) ranking high with the Rvs167 orthologs in the four yeast species but low with the *C*. *albicans* Rvs167-3 paralog, and the non-canonical Type I peptide #268 (ITH**R**LR**I**SI**P**GITGR) ranking high with *Ca*Rvs167-3 but low with Rvs167 orthologs (S4 Table). A moderate to strong interaction of the four Rvs167 SH3 domains with the Type II peptide #66 was observed whereas *Ca*Rvs167-3 did not show an interaction with peptide #66 above background levels. Conversely, *Ca*Rvs167-3 interacted strongly with peptide #268 while none of the Rvs167 orthologs showed an interaction. Neither of the two peptides interacted with *Ca*Rvs167-2, suggesting that this SH3 domain has a different binding specificity or may not be folded properly. Together, these results confirm the binding specificity of the Rvs167 proteins in the four yeast species towards Type II peptides and suggest that the *Ca*Rvs167-3 SH3 domain has a unique specificity.

### Predicting conservation of Myo5 and Rvs167 SH3 binding motifs in *S*. *cerevisiae* binding-partner orthologs

Next, we created a position-weighted matrix from the manually constructed alignments for the Myo5 and Rvs167 families to scan candidate binding partner sequences (see Materials and Methods) and identify SH3 binding sites, and consequently potential binding events conserved across orthologous SH3 domains.

#### The type I myosin interaction with Las17 and App1

Type I myosins share a conserved domain organization containing an N-terminal motor domain followed by tail homology domains 1 and 2 (TH1, TH2), an SH3 domain, and an acidic tail [33]. All four yeast species contain one homolog except for *S*. *cerevisiae*, which has two functionally redundant type I myosins, Myo3 and Myo5. The type I myosins have essential functions in endocytosis and actin cytoskeleton organization, and localize to cortical actin patches [34–38]. The SH3 domains of *S*. *pombe* and *S*.*cerevisiae* type I myosins induce Arp2/3-complex dependent actin polymerization *in vitro* [32,39–42], which requires their interaction with the conserved homologs of Wiskott-Aldrich syndrome protein (WASP) and WASP-interacting protein (WIP) [32,39].

**Fig 6:**
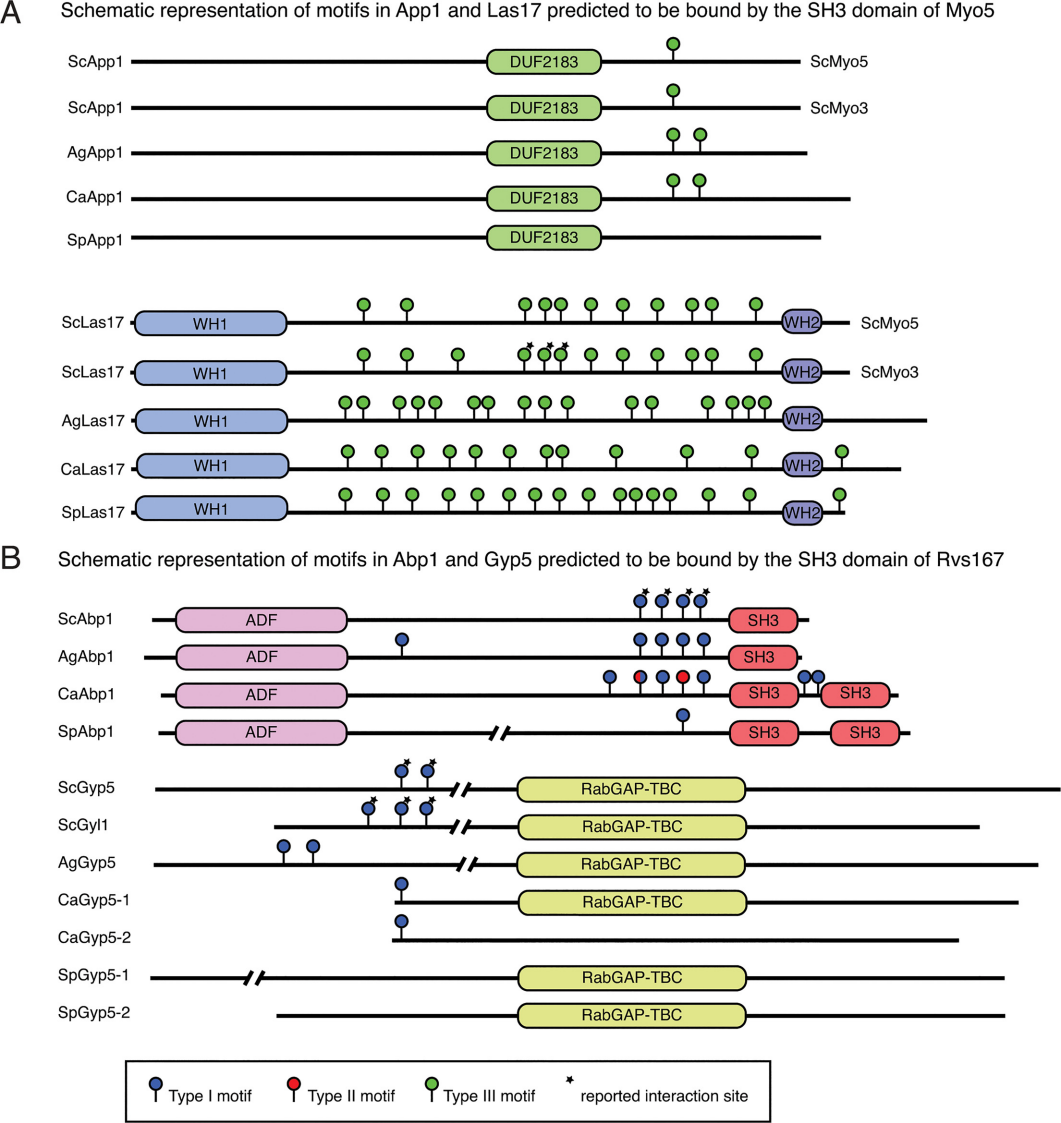
Scanning of homologous binding partner sequences with SPOT-derived PWMs reveals conservation of binding sites. (**A**) Sequence scanning of the *Sc*App1 and *Sc*Las17 homologs with a PWM of the Myo5 SH3 domains reveals that *Sp*App1 lost its Myo5 SH3 binding motif. The presence of multiple polyproline motifs in Las17 is conserved across all four yeasts. (**B**) Sequence scanning of the *Sc*Abp1 and *Sc*Gyp5 homologs shows that both *Sp*Gyp5-1 and SpGyp5-2 lost their Rvs167 SH3 binding motif. All significant hits are indicated by lollipops and colored according to the motif type (Type I, blue; Type II, red; TypeI/II, blue/red; Type III, green). Asterisks indicate previously reported interaction sites.

The confirmation that the type I myosins functionally interact with the *Sc*WIP homolog Vrp1 (Fig 5A) prompted us to analyze the interaction with WASP in more detail. The genomes of the four yeast species each encode one WASP homolog, Las17, which is required for normal cell growth, actin cytoskeleton organization, endocytosis and hyphal growth [40,43–45]. We used the PWM derived from the SH3 domain binding motifs (Fig 4) to scan the proteome sequences (see Materials and Methods) and identified the previously mapped binding sites for *Sc*Myo3 in *Sc*Las17, confirming our motif and approach [20] (Fig 6A). Furthermore, we predict several additional conserved potential binding sites for all SH3 domains in the central proline-rich region in agreement with the conserved motifs and functions of the type I myosins. *C*. *albicans* and *S*. *pombe* have an additional C-terminal motif, suggesting the presence of a new interaction site.

Furthermore, we looked at the App1 proteins predicted to interact with the SH3 domain of *S*. *cerevisiae* type I myosins [9]. The phosphatidate phosphatase App1 was shown in *S*. *cerevisiae* to localize to actin patches and interact with Rvs167 and Rvs161. Therefore, it most likely plays a role in endocytosis [46–48]. We identify one App1 homolog in each species and show conservation of a potential myosin-binding site in *S*. *cerevisiae*, *A*. *gossypii* and *C*. *albicans*. However, in *S*. *pombe* this motif is absent (Fig 6A), suggesting that *Sp*App1 may not interact with *Sp*Myo1.

On the one hand the type I myosins are conserved in their cellular localization and function. Also the binding motifs of the myosin SH3 domains are well conserved (Fig 4A), which is supported by their ability to induce actin polymerization in an *S*. *cerevisiae* extract. On the other hand the actual binding partners may differ as exemplified by the absence of the myosin binding motif in *Sp*App1 (Fig 6A), suggesting that a different mechanism may be operating in *S*. *pombe* as compared to the other yeast species analyzed.

#### The Rvs167 interaction with Abp1 and Gyp5

The Rvs167 proteins are characterized by the presence of an N-terminal BAR domain, a central domain of variable composition, and a C-terminal SH3 domain. *S*. *cerevisiae rvs167* deletion strains display a pleiotropic phenotype including growth sensitivity to salt, loss of bipolar bud site selection, and deficiencies in actin polarization and endocytosis (reviewed in [49]). More recently, Rvs167 has been implicated in polarized exocytosis [50]. In *C*. *albicans*, Rvs167 also plays an important role in endocytosis and actin polarization [24], but for the *S*. *pombe* homolog, called Hob1, current evidence suggests that it is not required for polarization of cortical actin and endocytosis [51,52].

To address conservation of binding specificity among the four yeast species, we selected three literature-validated interactors of *S*. *cerevisiae* Rvs167: the actin binding protein Abp1 as well as Gyp5 and Gyl1, two proteins that regulate Rab GTPases. The Rvs167 SH3 interaction site was mapped to a proline-rich region (PRR) N-terminal of the SH3 domains in Abp1 using *in vitro* binding and yeast two-hybrid assays [53,54]. Similarly, multiple independent approaches have revealed that the interaction of Gyp5 and Gyl1 with Rvs167 [31,55–57] requires the PRRs of Gyp5 and Gyl1 present in their N-terminal half [50]. We identified Abp1, Gyp5 and Gyl1 orthologs in the three other yeast species, except for a Gyl1 ortholog in *A*. *gossypii*, which appears to be missing. The protein sequences were scanned with the PWM for Rvs167 Type I and Type II motifs (Fig 6B). We identified the previously mapped Type II binding sites in the PRR of *S*. *cerevisiae* Abp1, Gyp5 and Gyl1, validating our approach. Conserved Type II binding sites were also predicted for the Rvs167 SH3 domains of the other three yeast species in Abp1, Gyl1 and Gyp5, with the exception of *S*. *pombe* Gyp5 proteins, which seem to lack the Type II (and Type I) motif. In *C*. *albicans* Abp1, two additional Type II binding sites N-terminal of the second SH3 domain are predicted as well as two Type I binding sites in the PRR. Together, these results suggest an overall conservation of Rvs167 binding sites in Abp1, which is consistent with a role of Rvs167 in actin polarization and endocytosis in all four yeast species. The absence of a binding site in *S*. *pombe* Gyp5 proteins may suggest that Rvs167 is not involved in polarized exocytosis in this yeast species. However, further cell biological experiments are required to address this.

### Overall conservation of the SH3-domain specificity landscape

**Fig 7:**
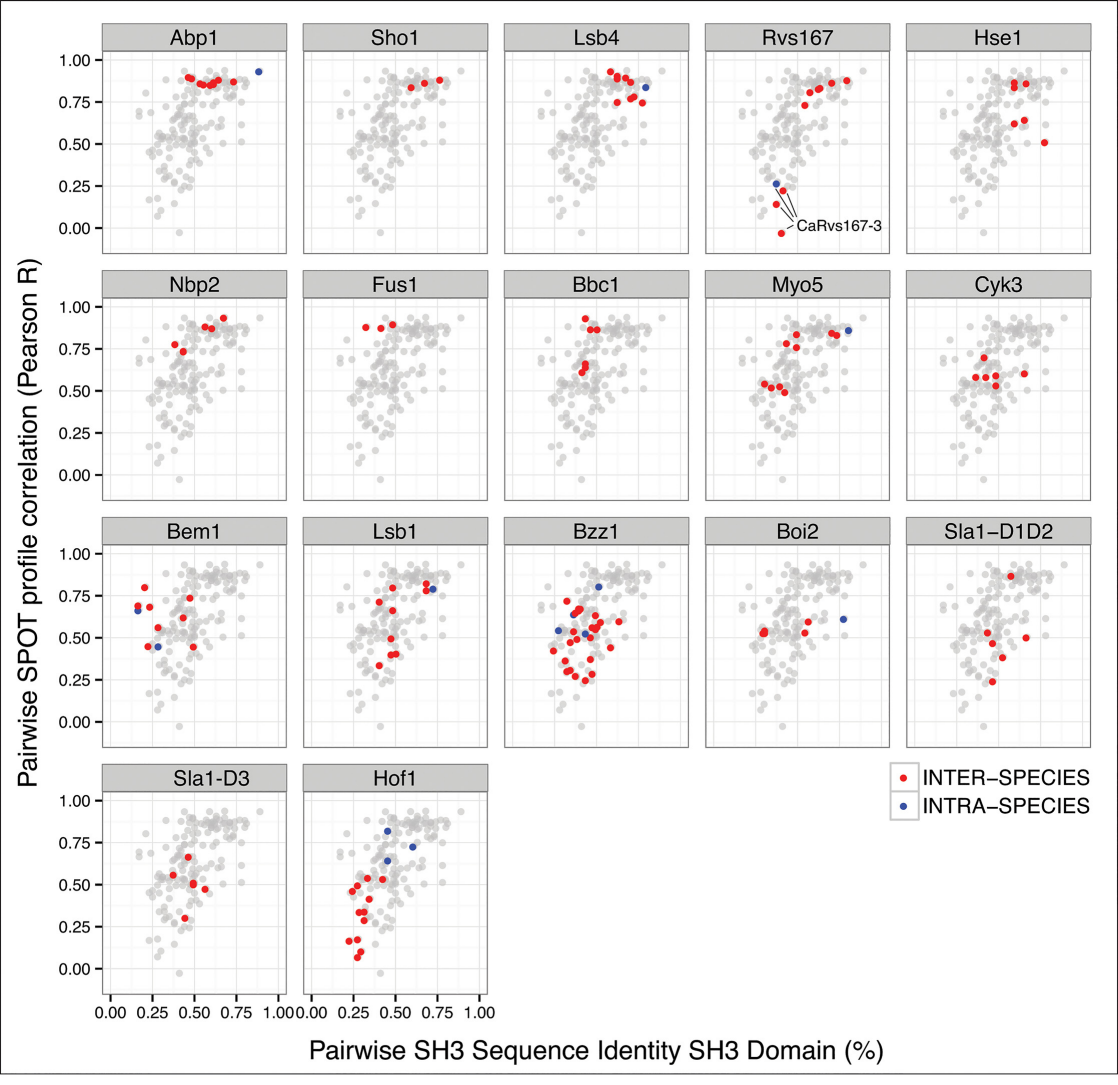
Correlation of sequence conservation and SPOT binding profile similarity per SH3 family. The relationship between sequence conservation and SPOT profile correlation is remarkably conserved for every pair of SH3 domains within an SH3 family. In contrast, *Ca*Rvs17-3 is a striking example of binding divergence, despite sequence conservation, within a single highly conserved SH3 family (lines in the Rvs167 panel). Paralogs and within-gene domain duplications are marked as intra-species (blue dots) while those between homologs in different species are marked as inter-species (red dots). SH3 families are ordered from high to low sequence and specificity conservation (left-to-right, top-to-bottom).

To evaluate how the SH3-domain specificity landscapes evolved in the four yeast species, we compared SH3 domain sequence conservation and SPOT profile correlation for every pair of SH3 domains within a single family (Fig 7). Overall, our data demonstrate a general correlation between sequence identity and binding specificity. For ~75% of the SH3 families analyzed, the specificity landscape was remarkably conserved over a large evolutionary distance of 400 Ma, with a high SH3 domain sequence identity predicting a conserved binding specificity. By contrast, the binding specificity was poorly conserved when the SH3 families showed a divergence in sequence identity, suggesting less evolutionary pressure on the SH3 domain function within these families. Interestingly, we did not observe a clear distinction between sequence and binding profile conservation patterns of intra-species versus inter-species comparisons between homologous SH3 domains, which is likely due to variable evolutionary pressure on these duplicated or homologous domains. The most striking observations are that, with respect to overall conservation in a family, the Hof1 paralogs have a remarkably conserved binding profile, whereas the binding profile of the CaRvs167-3 paralog changed dramatically, diverging from a Type II specificity typical for Rvs167 SH3 domains to a Type I-like specificity. This change in binding specificity cannot be fully explained by general divergence of the CaRvs167-3 SH3 sequence, but is likely supported by a conserved n-Src loop insertion in the Candida branch. However, a detailed molecular mechanism and a rationale for neo-functionalization of this transition remains to be elucidated.

In summary, we show that binding specificities obtained by probing a set of core binding-motif based peptides with orthologous SH3 domains from related organisms can be used for the prediction of potential SH3 domain interactors (Fig 6). We argue that the relevance of these findings goes beyond the improved understanding of SH3 domain network evolution, as it is likely that similar observations can be made for other common peptide recognition modules such as PDZ, SH2, and WW domains. As such, this study provides proof of principle for future analyses aimed at unraveling the complex specificity networks of peptide recognition modules in higher eukaryotes, including mammals.

## Materials and Methods

### SH3 domain selection and phylogenetic annotation

To identify all SH3 domain proteins in the four organisms and the homology relations between them we selected all proteins that contain an SH3 domain by searching the SMART database [58]. Based on predicted phylogenetic trees by PhylomeDB [59], MetaPhOrs [60] and Synergy [19], we assigned ortholog and paralog relationships among the different SH3 domain containing proteins across the four species.

### SH3 domain production

To create pGEX2tk-modified, two annealing oligonucleotides containing an NcoI and a NotI restriction site were ligated into BamHI/EcoRI digested pGEX2tk (GE Healthcare). The SH3 domain boundaries were defined as the union of the domain regions identified by BLAST [61], PFAM [62], and SMART [58]. DNA fragments encoding the identified domains were amplified from *S*. *cerevisiae*, *A*. *gossypii*, *C*. *albicans* and *S*. *pombe* genomic DNA by the polymerase chain reaction (PCR), cloned into the EMBL plasmid pETM30 and subcloned between the NcoI and NotI sites of pGEX2tk-modified, a vector designed for the expression and purification of SH3 domains fused to the C-terminus of glutathione S-transferase (GST).

*E*. *coli* BL21(DE3) was used to express the SH3 domains as GST-fusion proteins. Cells were lysed by sonication in 2 ml phosphate-buffered saline (PBS) supplemented with Protease Inhibitor Cocktail (complete, Roche). The extract was clarified for 15 min at 13,000 rpm and the domains purified using glutathione Sepharose 4B beads (GE Healthcare) according to the manufacturer’s instructions. The domains were eluted with reduced glutathione and dialyzed overnight against PBS containing 10% glycerol. Protein concentrations were determined using Bradford assay (Thermo Scientific Pierce Coomassie (Bradford) Protein Assay).

Bud14, Cdc25, and Bem1-SH3-2 in the four species and *Ca*Scp1 proved to be insoluble or hard to produce in the quantity required for the SPOT assay (200 μg) (S2 Table).

### SH3 SPOT peptide array selection, synthesis and quantification

Cellulose membrane-bound peptides were automatically prepared according to standard SPOT synthesis protocols using a Spot synthesizer (Abimed) as described in [63]. The software LISA (Jerini) was used for the generation of the peptide sequence files and all cysteines were replaced by serines to exclude false-positive spots. A conservative length of 15-mers was chosen to ensure efficient coupling steps during peptide synthesis in the absence of extensive HPLC and MS analyses of probes. The generated arrays of 15-mer peptides were synthesized on cellulose-(3-amino-2-hydroxy-propyl)-ether (CAPE) membranes. CAPE membranes were prepared from 18 × 28 cm Whatman 50 paper as described in detail [31].

The SPOT membrane was rinsed for 5 min with ethanol and washed three times with TBS (50 mM Tris/HCl, pH 7.6, 150 mM NaCl) for 10 min before blocking with blocking buffer (TBS, 1 x Blocking Buffer (Sigma B-6429), 0.15 M sucrose) for 3 hours. The SH3 domains were incubated at 10 μg/ml with the membrane overnight at 4°C in blocking buffer. The membrane was washed three times with TBS for 10 min. Immuno-detection was done by incubating the membrane for 2.5 hours with an anti-GST antibody (Sigma G-1160, 1 μg/ml), a secondary anti-mouse antibody HRP conjugate (Sigma A-5906, 1 μg/ml) and Luminol solution (Thermo Scientific # 34080). Pictures were taken using a Lumi Imager (Boehringer) and analyzed with the software Genespotter (Microdiscovery GmbH).

### Multiple sequence alignments of family members

All sequences were aligned with T-coffee (version 8.98) sequence alignment software [64] using the *accurate* mode. This mode combines information from Hidden Markov model (HMM) profiles (PSI-coffee) and three-dimensional information from structural templates (Expresso) with multiple sequence alignments from other alignment tools (ClustalW2) to create a highly informed meta-alignment. All multiple sequence alignments were rendered and edited with Jalview [65] to annotate the motifs. The template structures identified by T-coffee and detailed description by Fernandez-Ballester *et al*. [17] were used to visually inspect whether critical interface residues were spatially conserved.

### Genome scanning with PWMs

After manually aligning the top 95% peptides of each SH3 domain, we transformed the alignment into a 15 amino acid-wide position-weighted matrix (PWM), corresponding to the sequence length of the peptide probes, by computing the normalized observed frequency per amino acid for each position. Using a sliding window approach we computed a score for each 15-residue partial sequence in a potential binding sequence. A score for a subsequence is obtained by summing the substitution scores, using the PAM250 substitution matrix, of the observed amino acids to the amino acids in the PWM per residue position. To account for PWM specific score distributions, we computed for each score the probability of observing such a score given the PWM against a background distribution of 1000 randomly sampled 15-mers. These p-values were then corrected for multiple hypotheses testing by applying the Benjamini-Hochbach correction, which controls the false discovery rate (FDR) and converts p-values to q-values. Only subsequences with a q-value <0.0001 were retained as sequence matches to the PWM.

### Ex vivo actin polymerization

The coding sequences for the Myosin C-terminal tails were PCR amplified, cloned by restriction digestion into a pGEX plasmid and transformed into *E*.*coli* Rosetta cells (Merck). Cultures were grown at 30°C in LB (1% [w/v] tryptone, 0.5% [w/v] yeast extract, 1% [w/v] NaCl) broth medium containing 100 mg/l ampicillin and induced at an OD_600_ of 0.6 with 0.2 mM isopropyl β-D-thiogalactopyranoside (IPTG) for 3 h. Cells were lysed by sonication as described and GST fusion proteins were purified using glutathione Sepharose 4B beads (GE Healthcare) according to the manufacturer's instructions.

*S*. *cerevisiae* cells were grown overnight in YPD (1% [w/v] yeast extract, 2% [w/v] peptone, 2% [w/v] D(+)-glucose) to an OD_600_ of approximately 0.8, pelleted by centrifugation and resuspended in 1 ml of lysis buffer (PBS, 200 mM Sorbitol, Protease Inhibitor Cocktail (complete, Roche)). Upon a 1:1 (v/v) addition of glass beads, cells were vortexed 5 times for 30 s at 6.5 m/s using FastPrep 120 (MP Biomedicals) at 4°C. Cell debris were removed by centrifugation (13,000 rpm, 15 min) and protein concentration was determined by Bradford assay (20–30 mg/ml). The cleared lysate was aliquoted by snap freezing in liquid nitrogen and stored at -80°C.

The actin-polymerization assay was performed according to [32]. Briefly, 7 μl of total yeast protein extract were mixed with 1 μl of ATP-regenerating system (10 mg/ml creatine kinase, 10 mM ATP, 10 mM MgCl_2_, 400 mM creatine phosphate) and 1 μl of 0.4 g/l rhodamine-labeled actin (Cytoskeleton, Inc.). The polymerization reaction was initiated by adding 1 μl of 50% glutathione–Sepharose beads bound to the corresponding GST fusion protein. Samples were incubated at RT and visualized using fluorescence microscopy (Zeiss Axiovert 200M) after 15 min incubation. Latrunculin-A was added to a final concentration of 10 μM prior to the addition of the glutathione–Sepharose beads.

### Yeast two-hybrid assays

Double-stranded annealed oligonucleotides encoding the peptides of interest were cloned between NcoI and NotI sites of pGBKT7 (multicopy “bait” plasmid, Clontech). SH3 domains were cloned between NcoI and NotI sites of pYR035, which is pGADT7 (multicopy “prey” plasmid, Clontech) with a modified multiple cloning site allowing the insertion of NcoI-NotI fragments. Bait and prey plasmids were co-transformed into the *S*. *cerevisiae* yeast two-hybrid Gold strain (*Mata*, *trp1-901*, *leu2-3*, *112*, *ura3-52*, *his3-200*, *gal4Δ*, *gal80Δ*, *LYS2*::*GAL1*_*UAS*_ *-Gal1*_*TATA*_ *-His3*, *GAL2*_*UAS*_ *-Gal2*_*TATA*_ *-Ade2*, *URA3*::*MEL1*_*UAS*_ *-Mel*_*TATA*_ *AUR1-C MEL1*; Clontech) using a lithium acetate procedure [66]. Transformants were selected on minimal glucose plates (2% [w/v] D(+)-glucose, 0.67% [w/v] Yeast Nitrogen Base without amino acids [DIFCO], 2% [w/v] agar) lacking tryptophan and leucine. The strength of interaction was assessed by spotting ~10^4^ cells from an exponentially grown culture onto minimal agar plates without histidine (His^–^), without histidine and containing different amounts of 3-amino-1,2,4-triazole (3AT) (Sigma-Aldrich), or without adenine (Ade^–^). Growth on plates without adenine indicates a stronger interaction because the *ADE2* reporter gene has a weaker GAL promoter sequence than the *HIS3* reporter gene, while the inhibitor 3AT increases the stringency of the histidine selection. Plates were incubated for three days at 28°C before being photographed.

## Supporting Information

S1 Fig BAR-domain containing protein count in four yeast species. *Candida albicans* has approximately three times more BAR-domain containing proteins than each of the three other yeast species used in this study.
(PDF)

S2 Fig Structure-based sequence alignments of all SH3 domain families. Multiple sequence alignments of homologous SH3 domain sequences reveal conservation of the three SH3 interface motifs involved in ligand binding: the hydrophobic (red) and polar (blue) motifs in the RT loop and the WPY triad (green). In addition we report the RT and n-Src loop lengths based on alignments of structural models for each SH3 sequence.
(PDF)

S3 Fig Binding specificity logos for all SH3 domains characterized by the SH3-SPOT peptide assay. Manually curated alignments of the top 10 binding peptides for each SH3 domain were visualized by Weblogo as specificity profile logos and organized per family of SH3-domain containing protein homologs. Note that our specificity profile logos for the *Sp*Hof1 and *Sp*Hof1-2 SH3 domains are similar to the +*XLPXXP* motif observed by Ren and colleagues for these SH3 domains [21].
(PDF)

S1 File SH3 domain specificity mapped by SPOT membranes. Scheme showing the SH3-SPOT peptide assay layout with antibody and GST background controls alongside an image of an SH3-SPOT membrane incubated with a GST-only construct (**Figure A**). Images of all SH3-SPOT assays organized per family of SH3-domain containing protein homologs (**Figure B**). Clustered heat maps of correlation between normalized log2-scaled intensities organized per family of SH3-domain containing protein homologs. The heat maps are ranked from overall high within-family correlation (top-left) to lower within-family correlation (bottom-right) (**Figure C**).
(PDF)

S1 Table SH3 domain names and identifiers used in this study. (DOC)

S2 Table Cloned SH3 domains analyzed by SPOT. (XLSX)

S3 Table Averaged normalized SPOT intensities per SH3 domain. (XLSX)

S4 Table Peptide sequences used in the Yeast Two-Hybrid assay. (XLSX)
