# Supplementary figures and images for: Evolution of the SH3 Domain Specificity Landscape in Yeasts
Source: PLoS One. 2015 Jun 11;10(6):e0129229. doi: 10.1371/journal.pone.0129229 (PMC4466140; doi:10.1371/journal.pone.0129229)

Proteins with PFAM domain architecture

15  
10  
5  
0

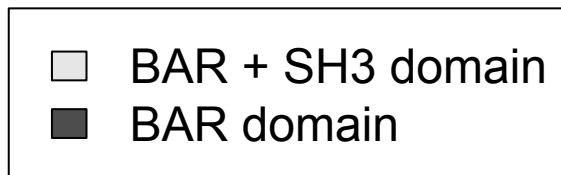

Ca

Sc

Ag

Sp

Species

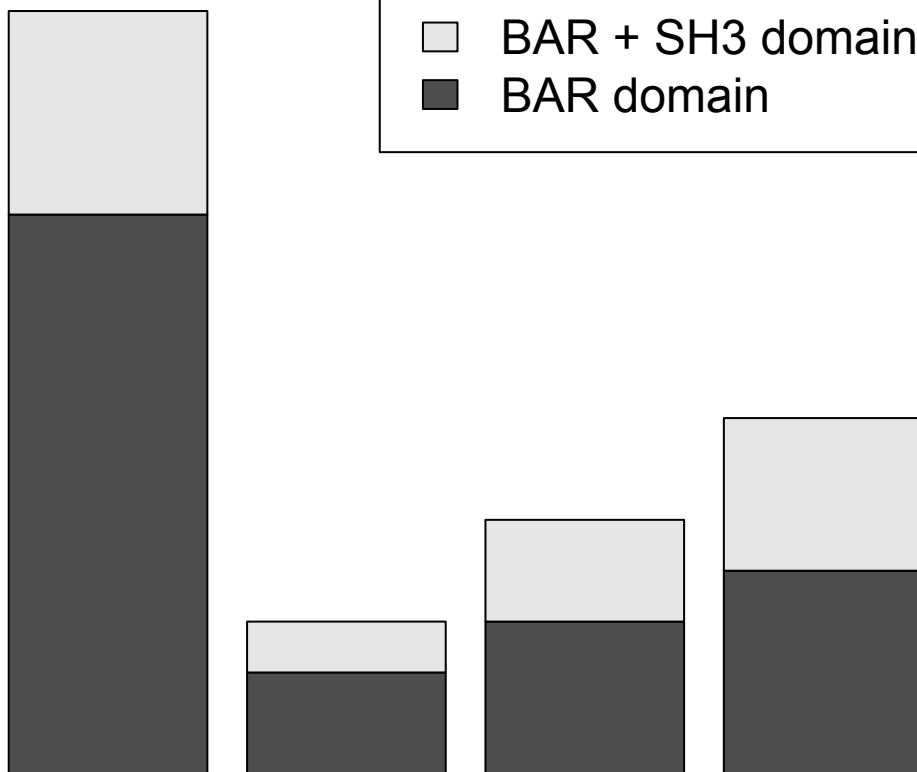

Supplement: S1 Fig — Candida albicans has approximately three times more BAR-domain containing proteins than each of the three other yeast species used in this study. (PDF) [file pone.0129229.s001.pdf]
