# Supplementary material for: Evolution of the SH3 Domain Specificity Landscape in Yeasts
Source: PLoS One. 2015 Jun 11;10(6):e0129229. doi: 10.1371/journal.pone.0129229 (PMC4466140; doi:10.1371/journal.pone.0129229)
Supplement: S2 Fig — Multiple sequence alignments of homologous SH3 domain sequences reveal conservation of the three SH3 interface motifs involved in ligand binding: the hydrophobic (red) and polar (blue) motifs in the RT loop and the WPY triad (green). In addition we report the RT and n-Src loop lengths based on alignments of structural models for each SH3 sequence. (PDF) [file pone.0129229.s002.pdf]

Domain	hydrophobic motif	polar motif	WPY triad	RT / n-Src loop lengths
**Abp1 family**				
AgAbp1-D1	YDY	ELTF	WPY	18 / 4
ScAbp1-D1	YDY	ELTF	WPY	18 / 4
CaAbp1-D2	YDY	EIGF	WPY	18 / 4
CaAbp1-D1	YDY	EIGF	WPY	18 / 4
SpAbp1-D2	YDY	ELSF	WPY	18 / 4
SpAbp1-D1	YDY	EIEL	WPY	18 / 4

```
                    14        24        34        44        54
AgAbp1-D1 PTAIAEYDYEAGEDNELTFEEGDVIVNIDFVDDDWWLGELQKTGQKGLFPSNYVELQQ
ScAbp1-D1 PWATAEYDYDAAEDNELTFVENDKIINIEFVDDDWWLGELEKDGSKGLFPSNYVSLGN
CaAbp1-D2 KTATAEYDYEKDEDNEIGFSEGDLIVEIEFVDDDWWQGKHSKTGEVGLFPANYVVLNE
CaAbp1-D1 TTATAEYDYEKDEDNEIGFSEGDLIIDIEFVDDDWWQGKHAKTGEVGLFPATYVSLNE
SpAbp1-D2 KSVKAIYDYQAQEDNELSFFEDEIIANVDCVDPNWWEGEC-H-GHRGLFPSNYVEE-I
SpAbp1-D1 TPAIVIYDYSPEEENEIELVENEQIQILEFVDDGWWLGENSK-GQQGLFPSNYVEITG
```

Domain	hydrophobic motif	polar motif	WPY triad	RT / n-Src loop lengths
**Bbc1 family**				
AgBbc1	YTY	DLNF	WPY	18 / 4
ScBbc1	FPY	DLSF	WPF	18 / 4
CaBbc1	FDY	DLNF	WPF	18 / 4
SpBbc1	YPY	DLEF	WPF	18 / 4

```
                10        20        30        40        50
AgBbc1 FDVIAKYTYESIHDGDLNFEDGQRIEVISIEDNQWYYGHFVDGAGNEQEGIFPKNYVEVAQ
ScBbc1 FKVVAQFPYKSDYEDDLNFEKDQEIIVTSVEDAEWYFGEYQDSNGDVIEGIFPKSFVAVQG
CaBbc1 MKVKAIFDYKSDYDEDLSFDAGTIINIISVENDEWYSGEYD---G--KQGMFPKNFVEELK
SpBbc1 TRVVALYPYRSSFSDDLEFDPGQVIDVVSNLDGDWYTGTYVDSDGNRKIGSFPKDFTEPAE
```

Domain	hydrophobic motif	polar motif	WPY triad	RT / n-Src loop lengths
**Bem1 family**				
ScBem1-D1	YSY	ELSF	WPY	18 / 3
CaBem1-D1	YDY	ELKF	WPY	18 / 13
SpBem1-D1	YDY	EVSF	WPH	18 / 5
AgBem1-D1	YTY	ELSF	WPY	18 / 3
AgBem1-D2	YDF	ELSA	WPF	18 / 4
CaBem1-D2	YEF	ELDI	WPY	18 / 4
ScBem1-D2	YDF	ELTT	WPF	18 / 4
SpBem1-D2	FDF	ELEA	WPF	18 / 4
CaBem1-2	YDF	EISV	WPY	18 / 4
AgBem1-2	QDY	FLNV	LPC	no model produced

```
                  37        47        57        67        77        87        97
ScBem1-D1 KVIKAKYSYQAQTSK---------ELSFMEGEFFYVSG-DEK---------DWYKASNPST-GKEGVVPKTYFEVFD
CaBem1-D1 KVIRALYDYEPQGPG---------ELKFFKGDFFHVLDDVDDELHKEAEANGWIEATNPMT-QLKGMVPISYFEIFD
AgBem1-D1 IILKALYTYHAQSPG---------ELSFNKGELFQVNG-EDG---------EWYEVTSTEG-GRKGMVPKSYFEQVS
SpBem1-D1 KVIRALYDYTARKAT---------EVSFAKGDFFHVIGREND--------KAWYEVCNPAA-GTRGFVPVSHFEEIG
AgBem1-D2 LYAIVLYDFKAEKSD---------ELSAFAGENLFICAHHNFE---------WFIAKPIGRLGGPGLVPVGFVSIID
CaBem1-D2 LYAVTLYEFKAERDD---------ELDIMPNENLIICAHHDYE---------WFIAKPINRLGGPGLVPVSYVKIID
ScBem1-D2 LYAIVLYDFKAEKAD---------ELTTYVGENLFICAHHNCE---------WFIAKPIGRLGGPGLVPVGFVSIID
SpBem1-D2 LFGIVQFDFAAERPD---------ELEAKAGEAIIIIARSNHE---------WLVAKPIGRLGGPGLIPLSFIQLRD
CaBem1-2  VILVTKYDFVAEGEN---------EISVNKGDLVKLIDRLGNG---------WVLVKYIDRVQPPGLIPASYVDIAV
AgBem1-2  LYYNVVQDYLPNSEEDAAGGRGGFLNVRRGDIVLHLEQAENN---------LVYVKLMNM-LGEGLVPARCLAVNR
```

Domain	hydrophobic motif	polar motif	WPY triad	RT / n-Src loop lengths
**Boi2 family**				
AgBoi2	NEY	DELD	WPF	18 / 8
ScBoi2	NEY	DELD	WPF	18 / 8
ScBoi1	NQY	DELN	WPF	18 / 8
CaBoi2	KQF	DELS	WPF	18 / 8
SpBoi2	HSF	DELP	WPH	18 / 8

```
              17        27        37        47        57
AgBoi2  GTYIAINEYSKRMEDELDMKPGDKIEVITDDQEYNDGWYFGRNLRTGEEGLYPKLFTQELS
ScBoi2  PMYIAINEYFKRMEDELDMKPGDKIKVITDDEEYKDGWYFGRNLRTNEEGLYPVVFTQKIT
ScBoi1  PLYIAVNQYSKRMEDELNMKPGDKIKVITDDGEYNDGWYYGRNLRTKEEGLYPAVFTKRIA
CaBoi2  DTYICIKQFNARLGDELSLKIGDKIQVLADDREYNDGWYMGKNLLTGEAGLYPKTFTQLIT
SpBoi2  RFVIALHSFPGKSSDELPLVEGRKYLLIKMDEEFGDGWWEGEDE-QGNRGIFPASHVELIS
```

Domain	hydrophobic motif	polar motif	WPY triad	RT / n-Src loop lengths
**Bud14 family**				
AgBud14	YAF	HCRL	WPI	18 / 5
CaBud14	YDF	HCTL	WPC	18 / 5
ScBud14	YAF	HCQL	WPI	18 / 5
SpBud14	RGF	QVDA	WPY	18 / 5

```
                 10        20        30        40        50        60        70        80
AgBud14  GKLYALYAFQGPDPSHCRLEPDESCILLNDQDSYWWLVKRC------------------------GDGKIGFAPAEILETFP
CaBud14  DKLYGLYDFSGPDPSHCTLSVDEPVYLINDEDNYWWLIRKLTKLERLKRMRLNGQEFQIDIESDEEDGKIGFVPAECLETHG
ScBud14  DKLYALYAFNGHDSSHCQLGQDEPCILLNDQDAYWWLVKRI------------------------TDGKIGFAPAEILETFP
SpBud14  NFVHAIRGFEATVEGQVDATKGDMMILLDDSNSYWWLVKMC------------------------KNLAIGYLPAEYIETPS
```

Domain	hydrophobic motif	polar motif	WPY triad	RT / n-Src loop lengths
**Bzz1 family**				
AgBzz1-D1	FPY	EVTI	WPY	18 / 5
ScBzz1-D1	YAY	EITI	WPY	18 / 5
AgBzz1-D2	YDY	EISI	WPY	18 / 5
CaBzz1-D1	YDY	ETTI	WPY	18 / 5
ScBzz1-D2	YAY	EISI	WPY	18 / 5
CaBzz1-D2	YDY	EISI	WPY	18 / 6
SpBzz1-D1	YDF	VITA	WPY	18 / 5
SpBzz1-D2	YAY	ELSI	WPY	18 / 5

```
                    14        24        34        44        54
AgBzz1_1  GGNRVLFPYTKQDEDEVTIAPGNSIVLLRQD-DGSGWVKIRNASTQQEGLVPSTYVEINE
ScBzz1_1  GKNKVLYAYVQKDDDEITITPGDKISLVARD-TGSGWTKINNDTTGETGLVPTTYIRISS
AgBzz1_2  -KVTAIYDYSAQDDDEISIRAGDVIKVLRGD-TGNGWTYGEV--NGSKGLFPSNYC---N
CaBzz1_1  IQARALYDYTAAGSDETTIVAGDQLSVIEED-DGSGWTMVNGP--QGQGLVPTSYIEITS
ScBzz1_2  RTMEAIYAYEAQGDDEISIDPGDIITVIRGD-DGSGWTYGECD--GLKGLFPTSYC---K
CaBzz1_2  QYLEALYDYTADGDDEISITAGDRIVLVQDDTDGSGWTEGELN--GQTGMFPTSYVKK-I
SpBzz1_1  VDGVVLYDFTGEHEGVITASEGQEFTLLEPD-DGSGWVRVKID--GTDGLIPASYVKLND
SpBzz1_2  S-VKALYAYTAQSDMELSIQEGDIIQVTNRN-AGNGWSEGIL--NGVTGQFPANYVTD-V
```

Domain	hydrophobic motif	polar motif	WPY triad	RT / n-Src loop lengths
**Cdc25 family**				
AgCdc25	CDF	HLPL	WPY	18 / 3
CaCdc25	YDF	HLPL	WPY	18 / 3
SpCdc25	SDY	FLKF	WPC	19 / 3
ScCdc25	YDF	LLSV	WPF	23 / 3
CaCdc25-2	HSF	CLSF	WPY	25 / 3
AgCdc25-2	HAF	CLSF	WPY	25 / 3

```
                20        30        40        50        60        70        80
AgCdc25    DVVRALCDFVPLK-------KQHLPLKAGDVVYVLSRHESGWCDGIIIEQTAKGNSTVTQCQRGWFPRTYTRSIR
CaCdc25    DTVIALYDFPGTQ-------SSHLPLNLGDTIHVLSKSATGWWDGVVMGN-------SGELQRGWFPHNYVRSVN
SpCdc25    FQTTAISDYENSS------NPSFLKFSAGDTIIVIEVLEDGWCDGICSE------------KRGWFPTSCIDSSK
ScCdc25    GIVVAAYDFNYPIKKD--SSSQLLSVQQGETIYILNKNSSGWWDGLVIDD------SNGKVNRGWFPQNFGRPLR
CaCdc25-2  FFIRALHSFDSSTLQSKSDASICLSFEKNDIAFLHTIDESGWGEVTLLE----------TLQTGWIPMNYFSLVV
AgCdc25-2  LFIVSIHAFDSQILENKEDATICLSFEKNELAFVHTVDESGWGEVTLVK----------SGRRGWVPFNYFADVV
```

Domain	hydrophobic motif	polar motif	WPY triad	RT / n-Src loop lengths
**Cyk3 family**				
AgCyk3	YGW	DLGF	WPY	18 / 3
ScCyk3	YGW	DLGF	WPF	18 / 3
CaCyk3	VSW	DLGF	WPY	18 / 4
SpCyk3	YAW	DLKF	WPF	18 / 4

```
                10        20        30        40        50
AgCyk3   FKVKAVYSWSGEHEQDLGFLESDIIEVTK-VKGNWLYGRLL-RNKKSGYFPVNYVQVLY
ScCyk3   FKVKARYGWSGQTKGDLGFLEGDIMEVTR-IAGSWFYGKLLRNKKCSGYFPHNFVILLE
CaCyk3   FKVKTIVSWAGEEEGDLGFMENEIVQVFSIVDESWWSGKLR-RNGAEGIFPKDYVTILE
SpCyk3   CMVRALYAWPGEREGDLKFTEGDLIECLSIGDGKWWIGRHI-NTNTQGIFPSNFVHCLD
```

Domain	hydrophobic motif	polar motif	WPY triad	RT / n-Src loop lengths
**Fus1 family**				
ScFus1	QDY	DEIR	WPC	18 / 5
AgFus1	KPF	DEIE	WPC	18 / 5
CaFus1	RSY	DELN	WPC	18 / 5

```
                 18        28        38        48        58        68        78
ScFus1   KTYTVIQDYEPRLTDEIRISLGEKVKILATHTDGWCLVEKCNTRKGTIH--VSVDDKRYLNEDRGIVPGDCLQEYD
AgFus1   DLCVVIKPFSPRLLDEIELNIDEQVRVLARHTDGWCLVEKCDSAKSADSDSDNISAPNYLNERRGIVPQMCLKHVF
CaFus1   SLYTVIRSYNKSLGDELNIEVGDKAVILEKHSDGWCKIRLVRMGKDYYN--------HQLSSDIGLVPKMCLQ--K
```

Domain	hydrophobic motif	polar motif	WPY triad	RT / n-Src loop lengths
**Hof1 family**				
ScHof1	YPL	LANF	WPF	19 / 4
AgHof1	YSF	ILYF	WPY	19 / 4
SpHof1-2	YDY	EISF	WPF	18 / 4
SpHof1-3	YAY	EIDL	WPY	18 / 4
SpHof1	YDY	EISF	WPF	18 / 4
CaHof1	YSF	ELYF	WPY	18 / 4

```
                   10        20        30        40        50        60
ScHof1     EYAKAMYPLIGNEAPGLANFHKGDYLLITEIVNKDWYKGEVYDNDRIDRNHRIGLIPYNFIQLLH
AgHof1     GYARAIYSFTEPNDNDILYFEMGDHLLLTEKLNTDWYIGEVHN-----GNGKQGLIPMNYVELLS
SpHof1-2   GYVIALYDYQAQI-PEEISFQKGDTLMVLRTQEDGWWDGEIINV----PNSKRGLFPSNFVQ--T
SpHof1-3   AYVRALYAYKATL-PSEIDLNVDDTLVVLNRQKDGWWKGLVVSP----TVGRIGLFPSNYIEELE
SpHof1     GYVSALYDYDAAI-PEEISFRKGDTIAVLKLYEDGWWEGFVVGE----DDHNRGQFPSNFVREIE
CaHof1     TKAIARYSFKPQT-EGELYFKKGWKMYIIHKQEDNWFVCELGDNCL-QSSGMIGLVPGNYIVEGD
```

Domain	hydrophobic motif	polar motif	WPY triad	RT / n-Src loop lengths
**Hse1 family**				
AgHse1	FDL	ELSF	WPY	18 / 4
ScHse1	YDL	ELSF	WPF	18 / 4
CaHse1	YDL	ELSF	WPY	18 / 4
SpHse1	YDF	ELSF	WPF	18 / 4

```
                17        27        37        56        66
AgHse1   RRVKAIFDLNASEPDELSFKKGDVITVIEQVYKDWWRGLLR-GKVGIFPVNYVGVCP
ScHse1   RRVRALYDLTTNEPDELSFRKGDVITVLEQVYRDWWKGALR-GNMGIFPLNYVTPIV
CaHse1   SKVRALYDLVSYEPDELSFRKGDVITVIESVYRDWWRGSLPSGKIGIFPLNYVTPIV
SpHse1   SRVRALYDFAATEQGELSFKKGDIILVLESVYKDWWKGSCK-NAVGIFPVNYVQRVV
```

Domain	hydrophobic motif	polar motif	WPY triad	RT / n-Src loop lengths
**Lsb1 family**				
AgLsb1	YAF	DLDF	WPY	18 / 4
ScLsb2	YQF	DLGL	WPY	18 / 4
ScLsb1	YDF	DLSL	WPY	18 / 4
CaLsb1	YDY	DLDL	WPY	18 / 4
SpLsb1	YDF	DLGF	WPY	18 / 4

```
                   12        22        32        42        52
AgLsb1   EYVEALYAFQAQQPGDLDFKVGEKIEVLEKPSPEWYKGR----CNGKVGMFPSNYVKPAF
ScLsb2   EYVEALYQFDPQQDGDLGLKPGDKVQLLEKLSPEWYKGS----CNGRTGIFPANYVKPAF
ScLsb1   EYVEALYDFEAQQDGDLSLKTGDKIQVLEKISPDWYRGK----SNNKIGIFPANYVKPAF
CaLsb1   GYCIATYDYKAQQAGDLDLSKGDKLAVVEHLSEDWWKGYKSDSSPEKTGVFPSNYVKIIS
SpLsb1   ERVLAMYDFPGPDAGDLGFHAGEVIIVLEHVNNDWWRGE----LNGKEGIFPSNYVRLLE
```

Domain	hydrophobic motif	polar motif	WPY triad	RT / n-Src loop lengths
**Lsb4 family**				
AgLsb4	YTF	DLPF	WPY	18 / 7
ScLsb3	YSF	DLPF	WPY	18 / 7
CaLsb4	YTF	DLPF	WPY	18 / 7
ScLsb4	YNF	DLAF	WPY	18 / 7
SpLsb4	YSF	DLSF	WPY	18 / 7

```
                  27        37        47        57        67
AgLsb4   PKAVALYTFKGEQKGDLPFRKGDVIMILKRTESQDDWWTGRINGQEGIFPANYVDLV
ScLsb3   PKAVALYSFAGEESGDLPFRKGDVITILKKSDSQNDWWTGRVNGREGIFPANYVELV
CaLsb4   TQAIALYTFKGEQSGDLPFKKGDVIDILKKTDTIDDWWTGRNNGLTGIFPANYVELI
ScLsb4   PTAVALYNFAGEQPGDLAFKKGDVITILKKSDSQNDWWTGRTNGKEGIFPANYVRVS
SpLsb4   NQARAMYSFAGEQPGDLSFQKGDIIDIVERSGSHDDWWTGRIGYREGIFPANYVKLS
```

Domain	hydrophobic motif	polar motif	WPY triad	RT / n-Src loop lengths
**Myo5 family**				
AgMyo5	YDF	ELPL	WPY	19 / 3
CaMyo5	YDF	EMTI	WPY	18 / 2
ScMyo5	YDF	ELPL	WPY	19 / 3
ScMyo3	FDY	SIPL	WPY	19 / 3
SpMyo1	FDF	ELPL	WPY	18 / 3

```
                  11        21        31        41        51
AgMyo5   PVYEAAFDFPGSGSPNEFPLKKGDRIY-VTRQEPSG-WSLAKALDGSKEGWVPTAYIVESK
ScMyo5   PMFEAAYDFPGSGSPSELPLKKGDVIY-ITREEPSG-WSLGKLLDGSKEGWVPTAYMKPHS
ScMyo3   PKFEAAYDFPGSGSSSELPLKKGDIVF-ISRDEPSG-WSLAKLLDGSKEGWVPTAYMTPYK
SpMyo1   DLYLALYDFAGR-SPNEMTIKK-DEIIEIVQKEPSG-WWLALKN--GAEGWVPATYVTEYK
CaMyo5   PTYKAMFDYDGS-VAGSIPLVK-DTIYYVT--QVNGKWGLVKTMDETKEGWSPIDYLKECS
```

Text

Domain	hydrophobic motif	polar motif	WPY triad	RT / n-Src loop lengths
**Nbp2 family**				
AgNbp2	YDF	ELEL	WPY	18 / 5
ScNbp2	YDF	ELRL	WPF	18 / 5
CaNbp2	FDF	EISL	WPY	18 / 5
SpNbp2	YDF	ELGF	WPF	18 / 5

```
                  24        34        44        54        64
AgNbp2   KHAWALYDFVPENDNELELQEGSIVYI-SYKHGQGWLVAEDSARTRTGLVPEEYVSILE
ScNbp2   QRAVALYDFEPENDNELRLAEGDIVFI-SYKHGQGWLVAENESGSKTGLVPEEFVSYIQ
CaNbp2   CKARAIFDFSAENDNEISLIEGQIIWI-SYRHGQGWLVAEDPISGENGLVPEEYVEIMQ
SpNbp2   ADAVALYDFEPLHDNELGFTTGQRLCILSESSD-GWLIAYDDASGRSGLVPETFVKLEV
```

Domain	hydrophobic motif	polar motif	WPY triad	RT / n-Src loop lengths
**Pex13 family**				
ScPex13	YDF	EVAL	WPY	19 / 10
CaPex13	YEF	EAPL	WPY	19 / 4
AgPex13	YDF	ECAL	WPY	19 / 10
SpPex13	YEF	EMSL	WPY	19 / 10

```
                   344       354       364       374       384       394
ScPex13   LEFARALYDFVPENPEMEVALKKGDLMAILSKKDPLGRDSDWWKVRTKNGNIGYIPYNYIEIIK
CaPex13   IQFARALYEFNPQNPQVEAPLEPKEIVAILDSRD------NWLRIRKRSGTMGWVPSNYVEVID
AgPex13   LEFARAIYDFTPENPRIECALKKGDLMAIISRQDPTGKESQWWKVRTKKGDVGYVPCNYIELIR
SpPex13   LEFCKADYEFMSRDPGVEMSLKKGDIIAILSKTDTQGNPCEWWQGRKRSGETGWFPSNYCSIIS
```

Domain	hydrophobic motif	polar motif	WPY triad	RT / n-Src loop lengths
**Rvs167 family**				
AgRvs167	YDF	DLSF	WPY	18 / 5
CaRvs167	YDY	DLTF	WPY	18 / 5
ScRvs167	YDY	DLSF	WPY	18 / 6
SpRvs167	YDY	DLSF	WPY	18 / 6
CaRvs167-2	HDF	DLRF	WPY	18 / 3
CaRvs167-3	YDF	DLSF	WPY	18 / 14
AgRvs167-2	YDY	YLSI	WPY	20 / 4
SpScp2	YSF	ELKL	WPY	18 / 4
SpScp1	YSF	ELDL	WPY	18 / 4

```
                   15        25        35        45        55        65        75
AgRvs167     ETCIALYDFAAQA--DGDLSFPVNAIIEILDRSDA---------AGWWTGRY-------NGREGLFPANYVALKH
CaRvs167     PTCTALYDYTAQA--QGDLTFPAGAVIEIIQRTED--------ANGWWTGKY-------NGQTGVFPGNYV---Q
ScRvs167     ETVTALYDYQAQA--AGDLSFPAGAVIEIVQRTPD--------VNEWWTGRY-------NGQQGVFPGNYVQLNK
SpRvs167     EHVVALYDYAAQA--AGDLSFHAGDRIEVVSRTDN--------QNEWWIGRL-------NGAQGQFPGNYVQL-E
CaRvs167-2   SYCYALHDFAGQE--ELDLRFSKGDKIKILVGN-----------GTWWERQL-------NGKIGQFPSNYVQL-I
CaRvs167-3   VLKTAKYDFSGYE--PGDLSFKQGDNIEILLDFQNIDTLYHSDELNWLIGVSRI--DDESYRIGFVPNNYVE---
AgRvs167-2   VYATALYDYDPQFNDPQYLSIKKGDIIQVITQSK----------NGWWYGDLL-----RTKTKGLFPQSYVQVQD
SpScp2       KVVRMKYSFEPET--ENELKLKKGDLLLVLKEID----------EGWWVGEKLGEDGVFTGNTGMFPSNYCVPAH
SpScp1       VFVKAIYSFTGRN--EKELDLHTGDVIQVSEQLG----------PDWYMGEKVNSKAEKLGNSGMFPVNYCTRIY
```

Domain	hydrophobic motif	polar motif	WPY triad	RT / n-Src loop lengths
**Sla1 family**				
AgSla1-D1	YAY	ELAL	WPY	18 / 6
AgSla1-D2	YDY	ELVF	WPY	20 / 5
AgSla1-D3	YNF	ELTV	WPY	18 / 6
CaSla1-D1	YDY	ELNI	WPY	18 / 6
CaSla1-D2	YDY	ELSF	WPY	18 / 5
CaSla1-D3	YDF	ELDC	WPF	18 / 6
ScSla1-D1	YAY	ELAI	WPY	18 / 6
ScSla1-D2	YDY	ELTF	WPY	20 / 5
ScSla1-D3	YDF	ELTI	WPF	18 / 6
SpSla1-D1	YSY	EISI	WPY	28 / 5
SpSla1-D2	YDY	EISF	WPY	18 / 5
SpSla1-D3	YDF	ELTV	WPF	18 / 6

```
                   10        20        30        40        50        60        70
AgSla1-D1   GVYKAVYAYEP--Q----------NEEELALEEDELLYLLQKSEVDDWWTVKKRVL---GGDMDEPVGLVPNNYIEEAP
AgSla1-D2   GRVRSVYDYEEAQN----------PDEELVFREGDEFDVYDDR-DADWVLVRKRA--------DGSVGFAPGNYVEKVG
AgSla1-D3   KRGIIKYNFFG--E----------SSDELTVKEGDVVYILNDKKSADWWMCEIIG--------TSRQGVIPAQFIDVQP
CaSla1-D1   GVYKALYDYAA--Q----------AEEELNIKQNDLLYLLEKSDIDDWWKVKKRVVATGEEIVDEPSGLVPSTYIEEAP
CaSla1-D2   KTATALYDYDK--Q----------TEEELSFNENDKFNVFDLN-DPDWILVGDLA--------KEKFGFVPSNYIQLDS
CaSla1-D3   KIGRLLYDFEV--Q----------GDDELDCKEGDEVYIIDQKKSKDWWMVENIA--------TRRQGVVPSTYIEIIS
ScSla1-D1   G-YRAVYAYEP--Q----------TPEELAIQEDDLLYLLQKSDIDDWWTVKKRVI---GSDSEEPVGLVPSTYIEEAP
ScSla1-D2   KKVRAIYDYEQVQN----------ADEELTFHENDVFDVFDDK-DADWLLVKSTV--------SNEFGFIPGNYVEPEN
ScSla1-D3   KRGIVQYDFMA--E----------SQDELTIKSGDKVYILDDKKSKDWWMCQLVD--------SGKSGLVPAQFIEPVR
SpSla1-D1   GIYKVLYSYEP--QEINPGEEIPENEREISIVEDEIVCLLEKG-EDDWYLVKRNVN---SNDDDEEIGIVPSNYITEAE
SpSla1-D2   TKMKALYDYTQ--Q----------SVDEISFQADQTLDCYGDT-DSDWILVGF----------NNNFGLAPRNYVEGMD
SpSla1-D3   KMGTVLYDFIA--E----------AADELTVKANMRVVIVNDTASSDWWKCSV----------DGKEGVVPSNFIKPDT
```

Domain	hydrophobic motif	polar motif	WPY triad	RT / n-Src loop lengths
**Sho1 family**				
AgSho1	YAY	EISF	WPY	20 / 5
ScSho1	YPY	EISF	WPY	20 / 5
CaSho1	YSY	EISF	WPY	20 / 5

```
                 15        25         36         46         57
AgSho1   YTAKALYAYEADASDAYEISFQQGEILRVGDIEGRWWKAKKANGETGIIPSNYVELVD
ScSho1   YKAKALYPYDADDDDAYEISFEQNEILQVSDIEGRWWKARRANGETGIIPSNYVQLID
CaSho1   YKAKALYSYDANPDDINEISFVKDEILEVDDIDGKWWQARRANGQVGICPSNYVKLLD
```
