# Supplementary material for: Evolution of the SH3 Domain Specificity Landscape in Yeasts
Source: PLoS One. 2015 Jun 11;10(6):e0129229. doi: 10.1371/journal.pone.0129229 (PMC4466140; doi:10.1371/journal.pone.0129229)

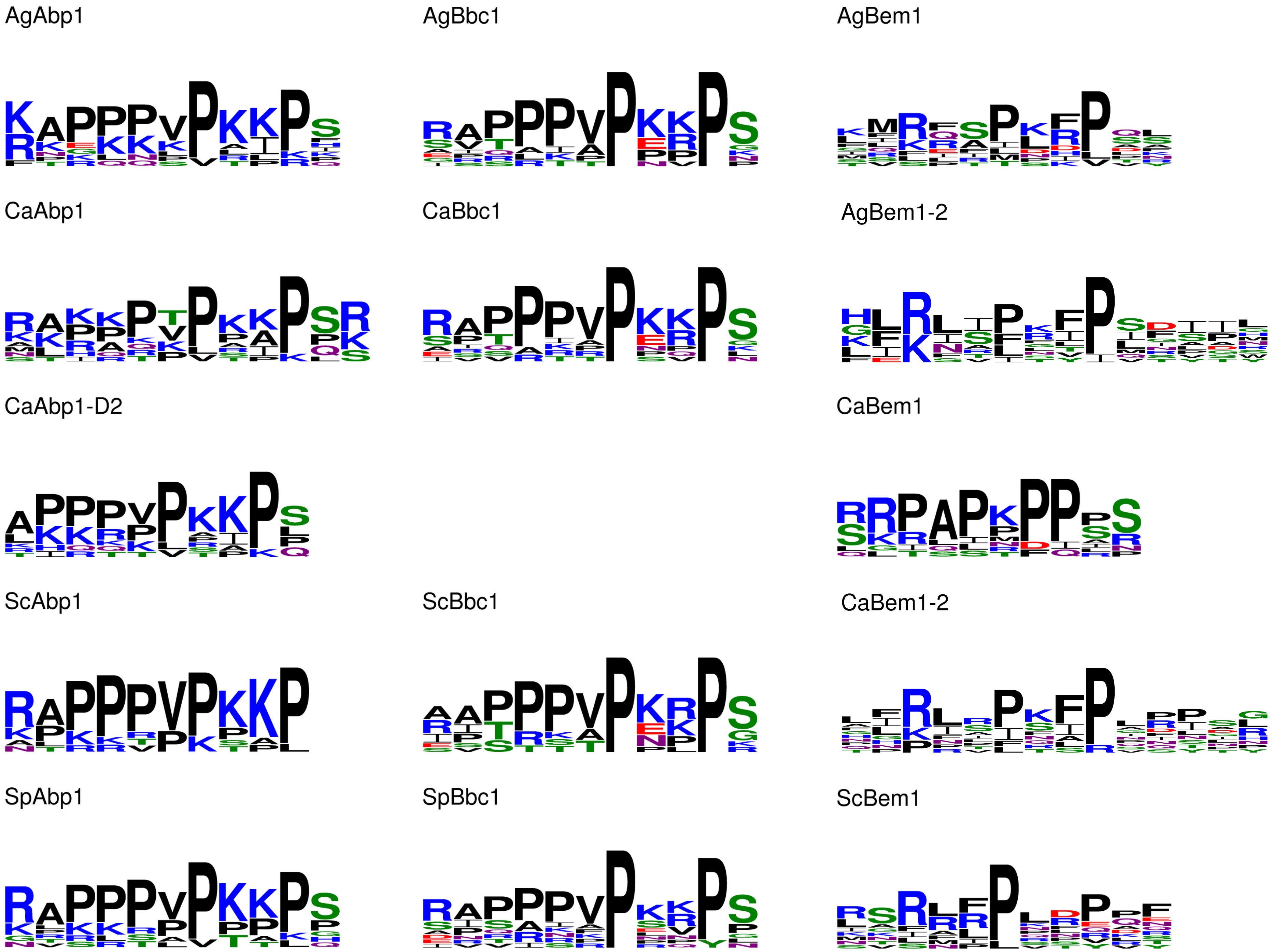
AgAbp1
AgBbc1
AgBem1
CaAbp1
CaBbc1
AgBem1-2
CaAbp1-D2
CaBem1
ScAbp1
ScBbc1
CaBem1-2
SpAbp1
SpBbc1
ScBem1

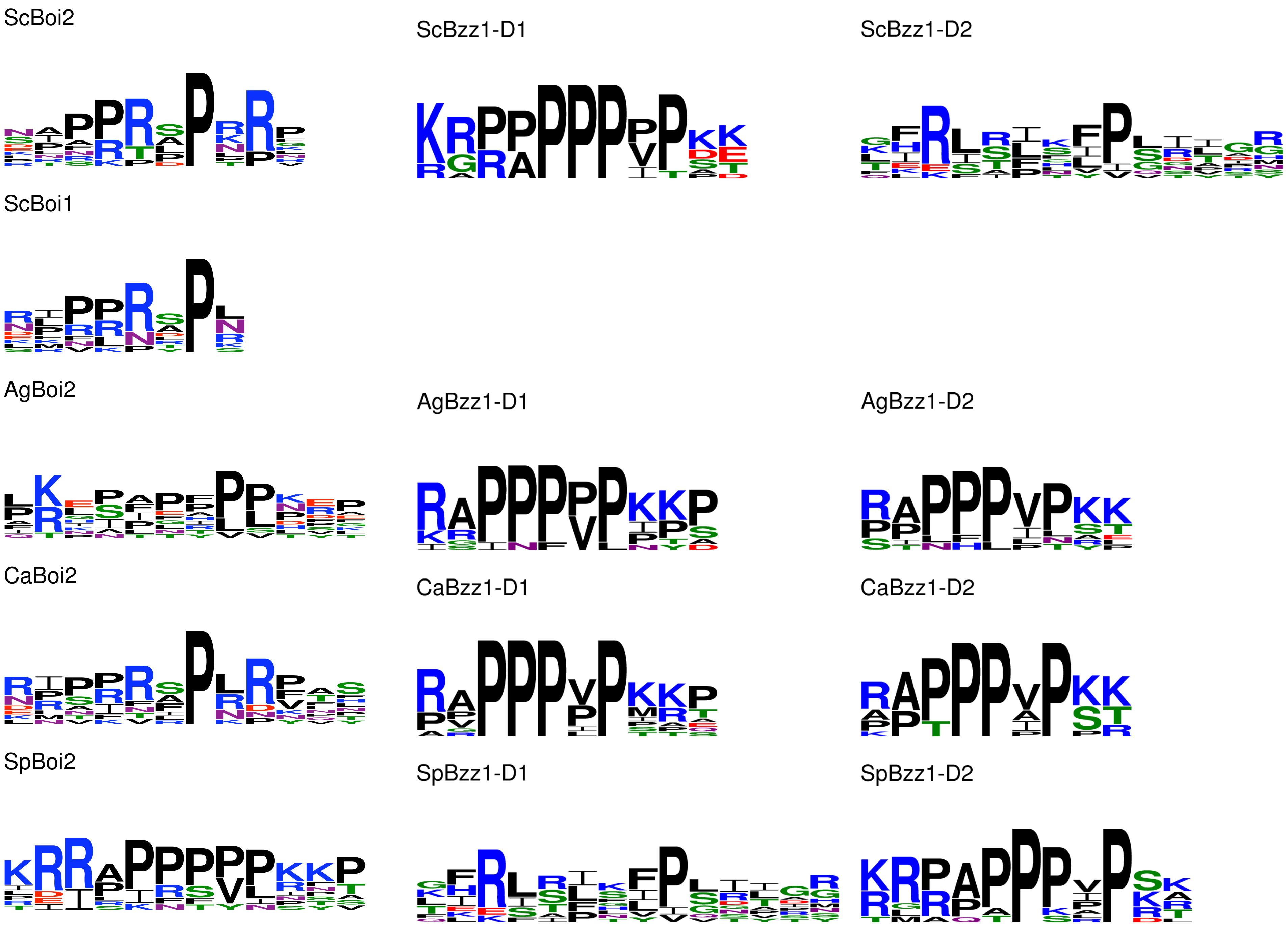
ScBoi2
ScBzz1-D1
ScBzz1-D2
ScBoi1
AgBoi2
AgBzz1-D1
AgBzz1-D2
CaBoi2
CaBzz1-D1
CaBzz1-D2
SpBoi2
SpBzz1-D1
SpBzz1-D2

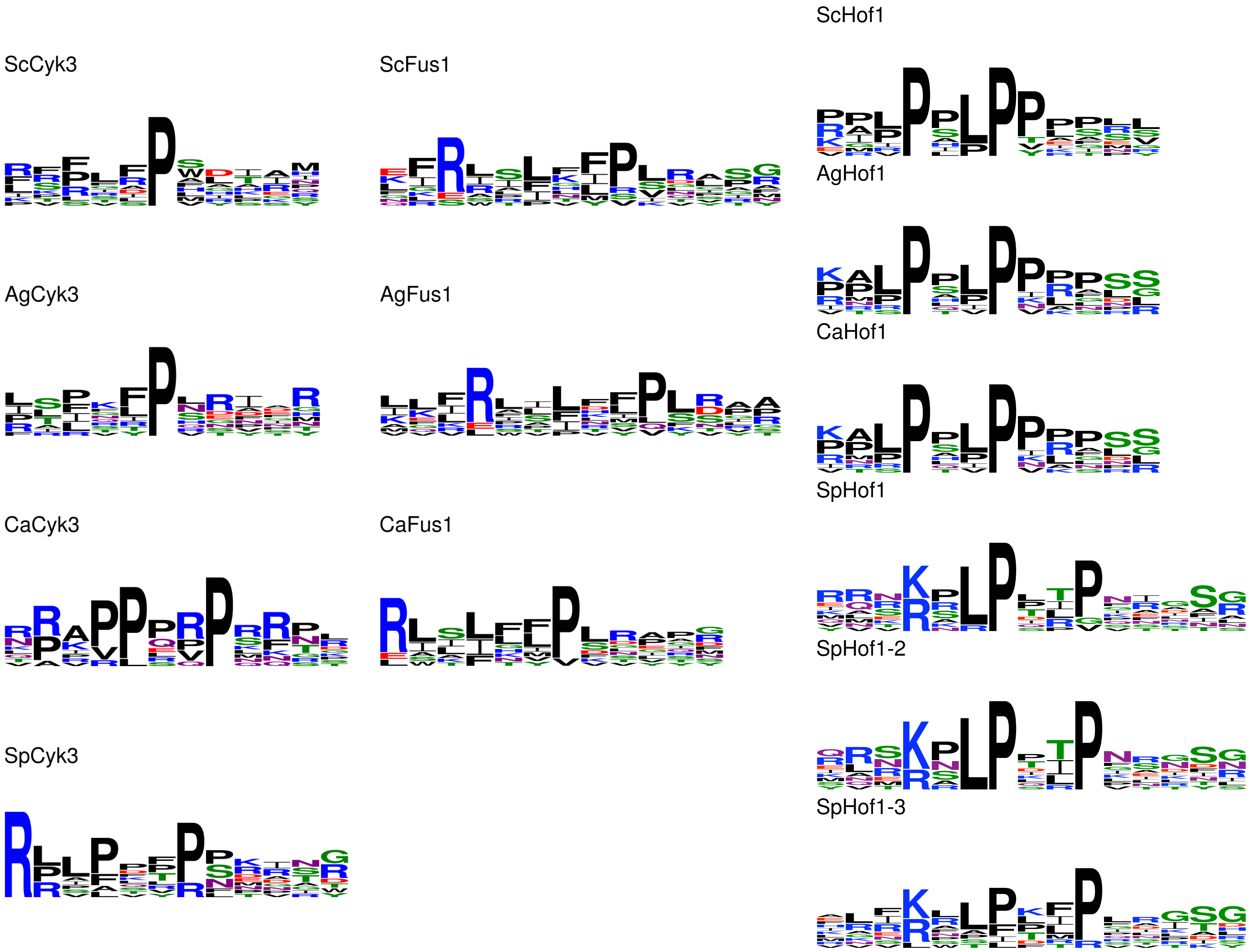
ScCyk3
ScFus1
ScHof1
AgCyk3
AgFus1
AgHof1
CaCyk3
CaFus1
CaHof1
SpCyk3
SpHof1
SpHof1-2
SpHof1-3

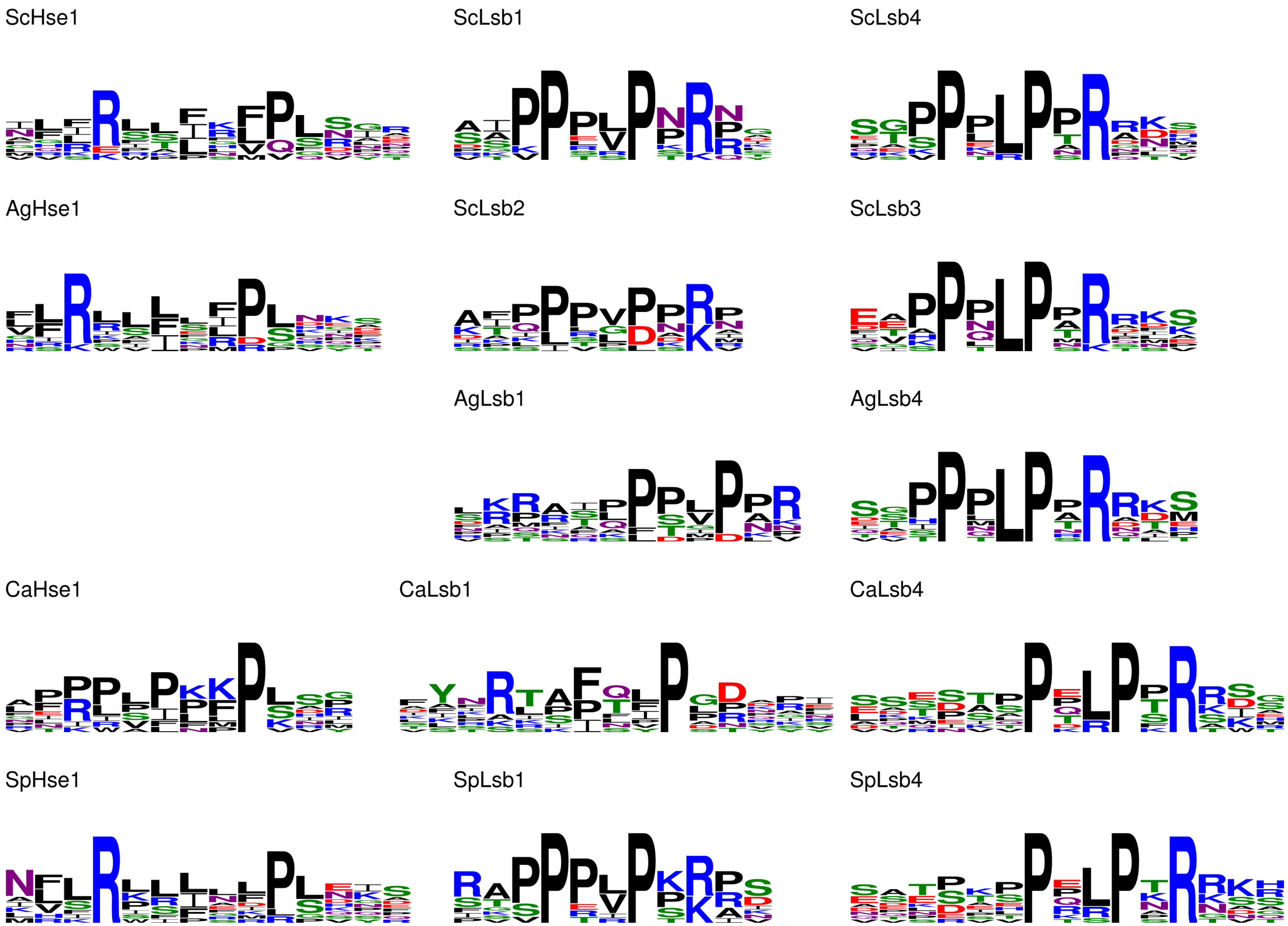
ScHse1
ScLsb1
ScLsb4
AgHse1
ScLsb2
ScLsb3
AgLsb1
AgLsb4
CaHse1
CaLsb1
CaLsb4
SpHse1
SpLsb1
SpLsb4

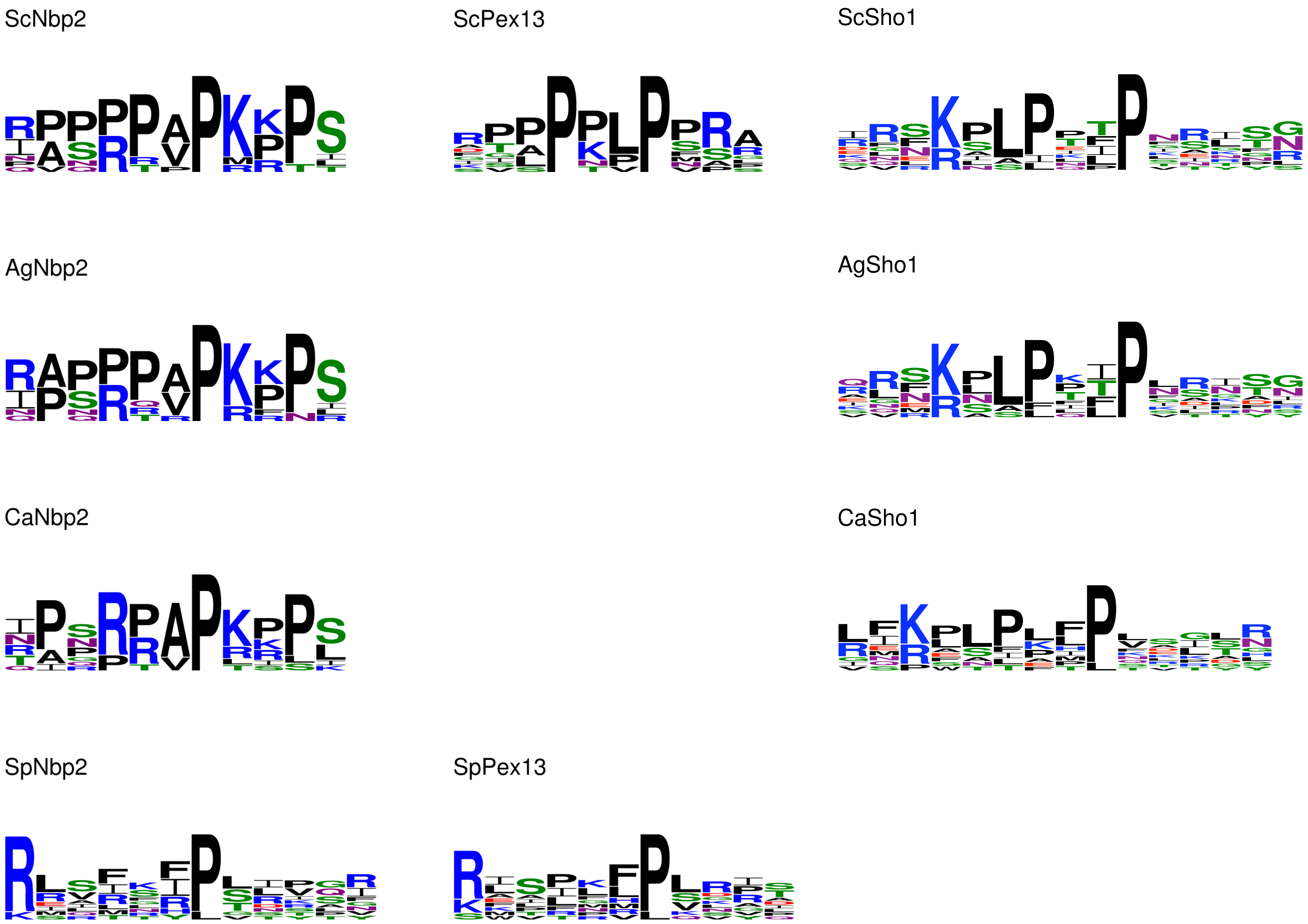
ScNbp2
ScPex13
ScSho1
AgNbp2
AgSho1
CaNbp2
CaSho1
SpNbp2
SpPex13

ScSla1-D1-D2

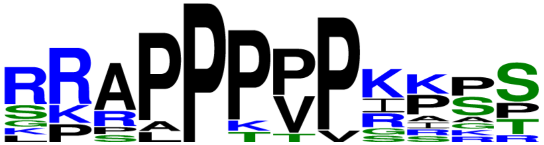

ScSla1-D3

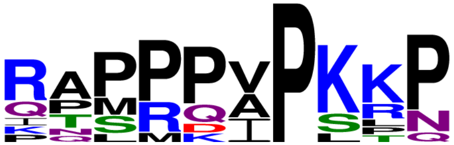

AgSla1-D1-D2

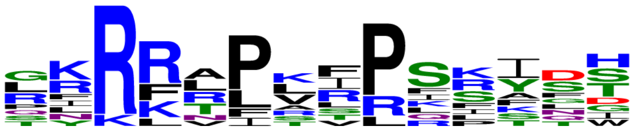

AgSla1-D3

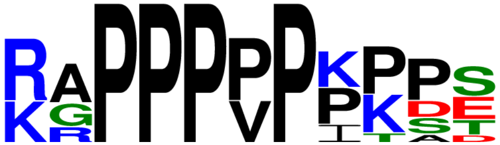

CaSla1-D1-D2

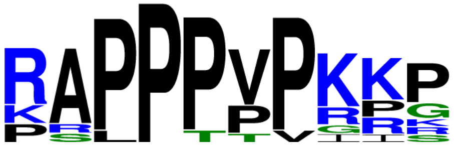

CaSla1-D3

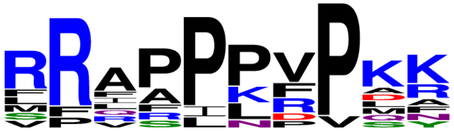

SpSla1-D1-D2

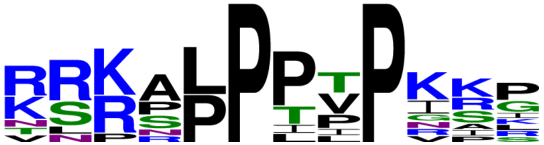

SpSla1-D3

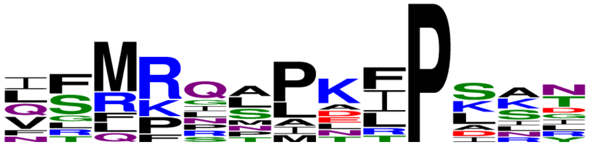

Supplement: S3 Fig — Manually curated alignments of the top 10 binding peptides for each SH3 domain were visualized by Weblogo as specificity profile logos and organized per family of SH3-domain containing protein homologs. Note that our specificity profile logos for the SpHof1 and SpHof1-2 SH3 domains are similar to the +XLPXXP motif observed by Ren and colleagues for these SH3 domains [21]. (PDF) [file pone.0129229.s003.pdf]
