# Supplementary material for: Evolution of the SH3 Domain Specificity Landscape in Yeasts
Source: PLoS One. 2015 Jun 11;10(6):e0129229. doi: 10.1371/journal.pone.0129229 (PMC4466140; doi:10.1371/journal.pone.0129229)
Supplement: S1 File — Scheme showing the SH3-SPOT peptide assay layout with antibody and GST background controls alongside an image of an SH3-SPOT membrane incubated with a GST-only construct (Figure A). Images of all SH3-SPOT assays organized per family of SH3-domain containing protein homologs (Figure B). Clustered heat maps of correlation between normalized log2-scaled intensities organized per family of SH3-domain containing protein homologs. The heat maps are ranked from overall high within-family correlation (top-left) to lower within-family correlation (bottom-right) (Figure C). (PDF) [file pone.0129229.s004.pdf]

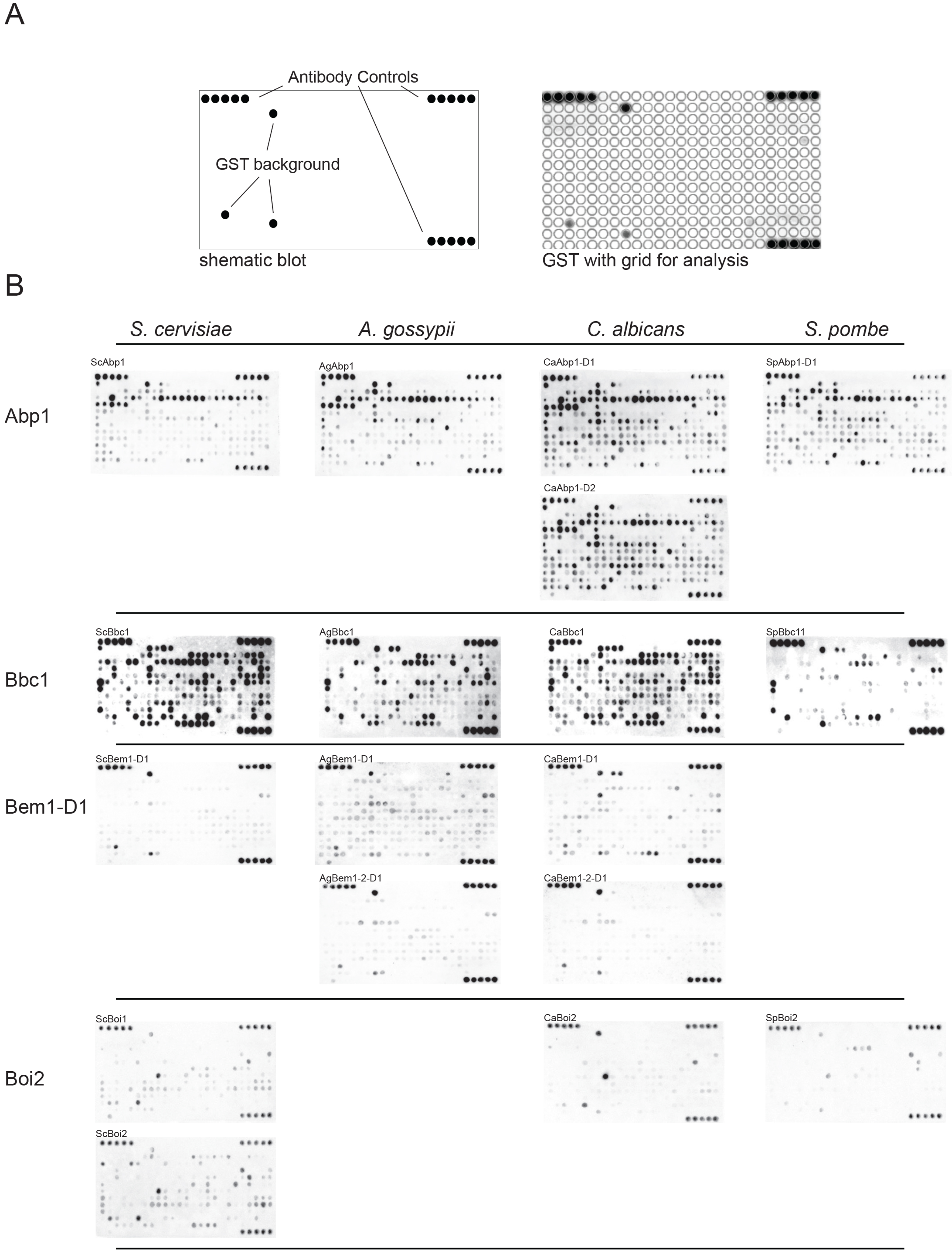
A
Antibody Controls
GST background
shematic blot
GST with grid for analysis
B
S. cervisiae
A. gossypii
C. albicans
S. pombe
Abp1
ScAbp1
AgAbp1
CaAbp1-D1
SpAbp1-D1
CaAbp1-D2
Bbc1
ScBbc1
AgBbc1
CaBbc1
SpBbc11
Bem1-D1
ScBem1-D1
AgBem1-D1
CaBem1-D1
AgBem1-2-D1
CaBem1-2-D1
Boi2
ScBoi1
CaBoi2
SpBoi2
ScBoi2

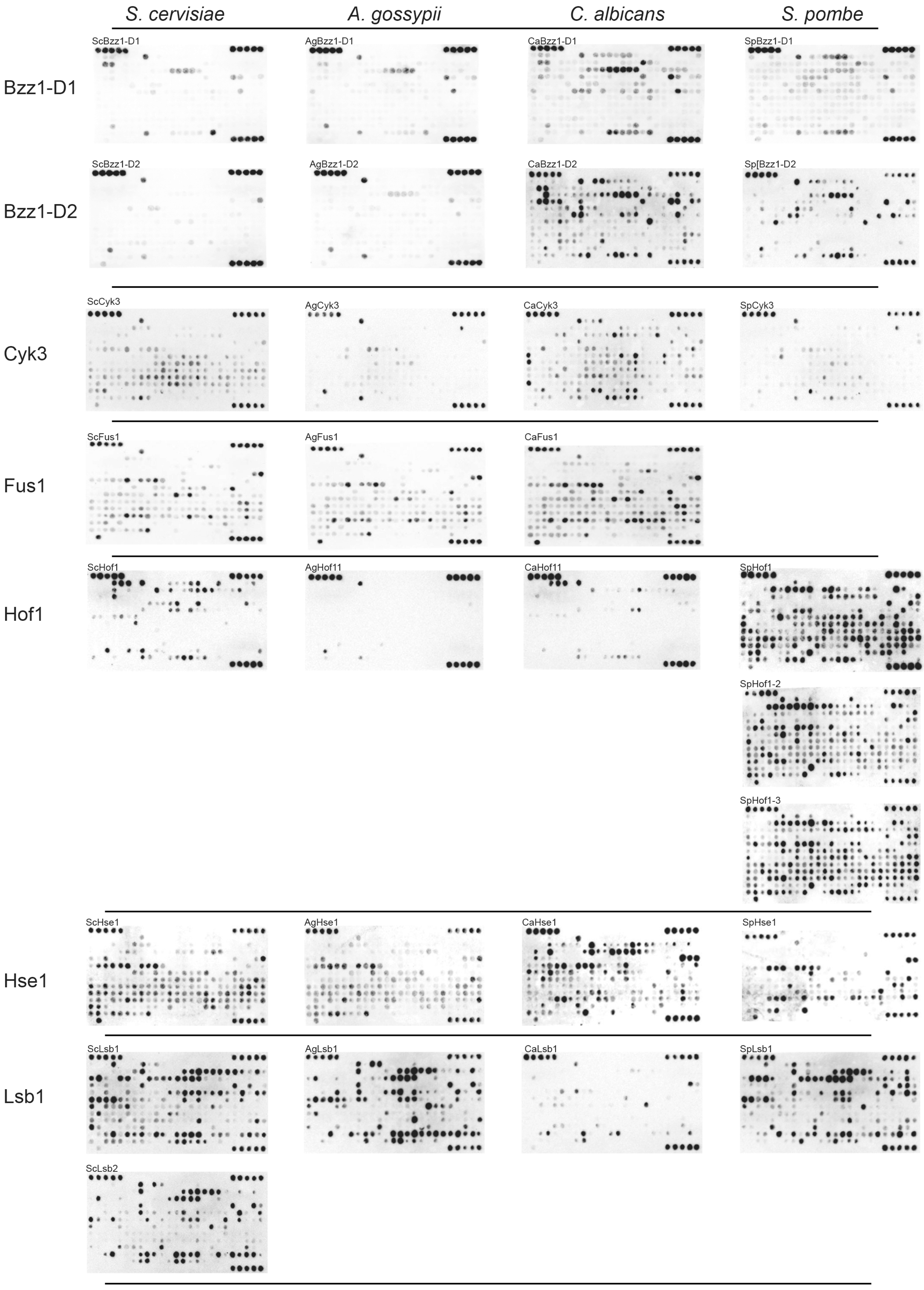
S. cervisiae
A. gossypii
C. albicans
S. pombe
Bzz1-D1
ScBzz1-D1
AgBzz1-D1
CaBzz1-D1
SpBzz1-D1
Bzz1-D2
ScBzz1-D2
AgBzz1-D2
CaBzz1-D2
Sp[Bzz1-D2
Cyk3
ScCyk3
AgCyk3
CaCyk3
SpCyk3
Fus1
ScFus1
AgFus1
CaFus1
Hof1
ScHof1
AgHof11
CaHof11
SpHof1
SpHof1-2
SpHof1-3
Hse1
ScHse1
AgHse1
CaHse1
SpHse1
Lsb1
ScLsb1
AgLsb1
CaLsb1
SpLsb1
ScLsb2

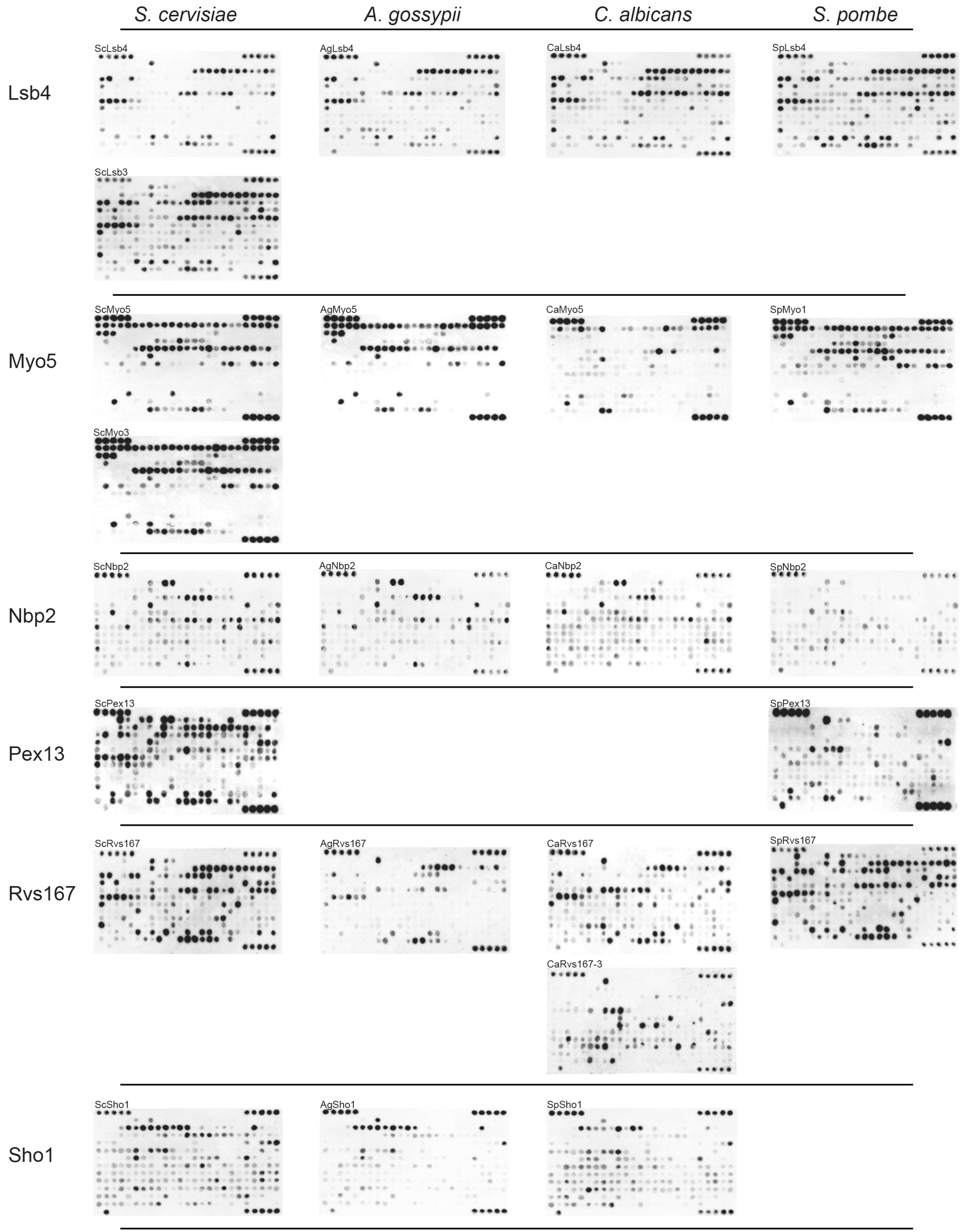
S. cervisiae
A. gossypii
C. albicans
S. pombe
Lsb4
ScLsb4
AgLsb4
CaLsb4
SpLsb4
ScLsb3
Myo5
ScMyo5
AgMyo5
CaMyo5
SpMyo1
ScMyo3
Nbp2
ScNbp2
AgNbp2
CaNbp2
SpNbp2
Pex13
ScPex13
SpPex13
Rvs167
ScRvs167
AgRvs167
CaRvs167
SpRvs167
CaRvs167-3
Sho1
ScSho1
AgSho1
SpSho1

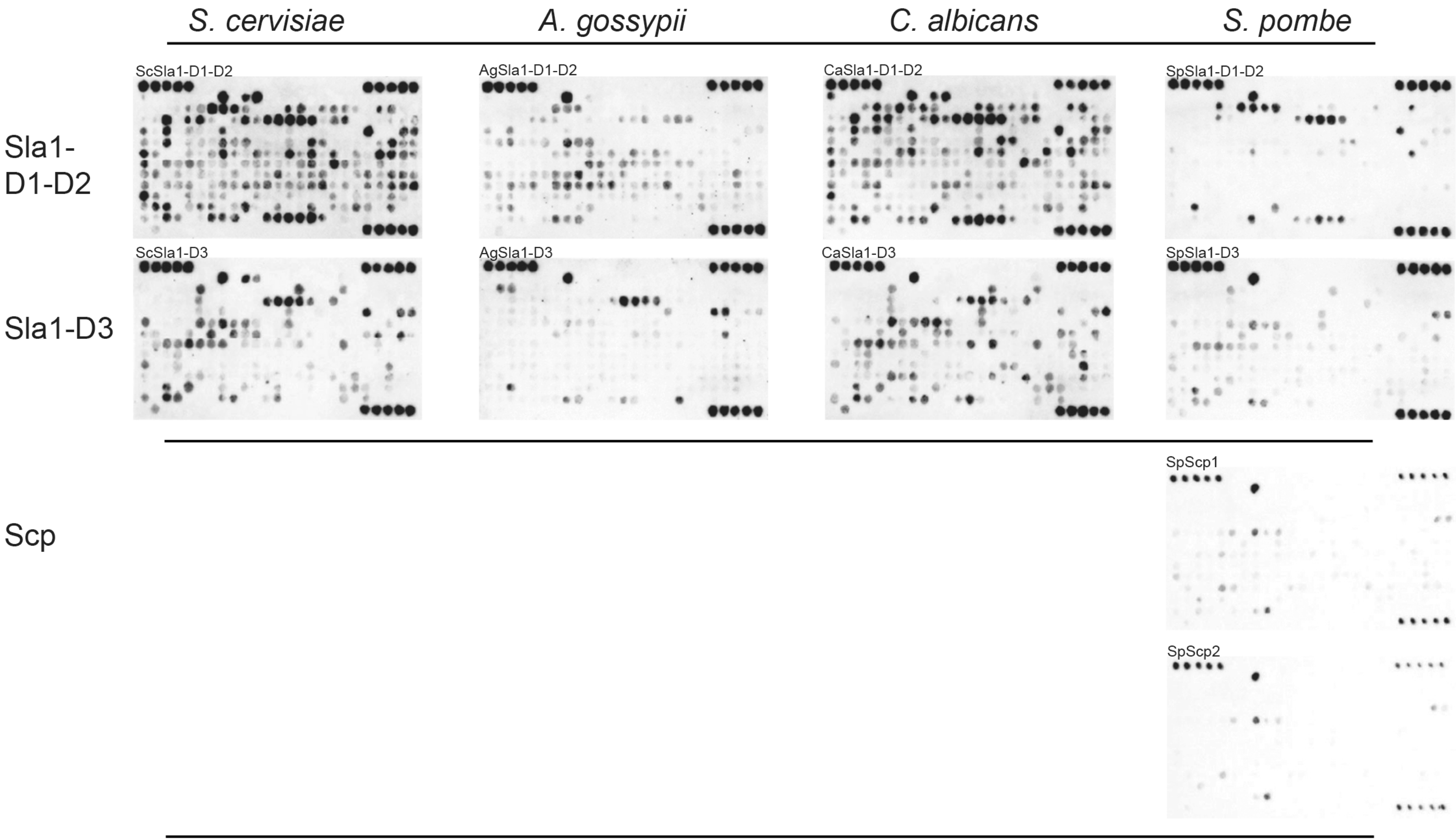
S. cervisiae
A. gossypii
C. albicans
S. pombe
Sla1-D1-D2
Sla1-D3
Scp
ScSla1-D1-D2
AgSla1-D1-D2
CaSla1-D1-D2
SpSla1-D1-D2
ScSla1-D3
AgSla1-D3
CaSla1-D3
SpSla1-D3
SpScp1
SpScp2

C

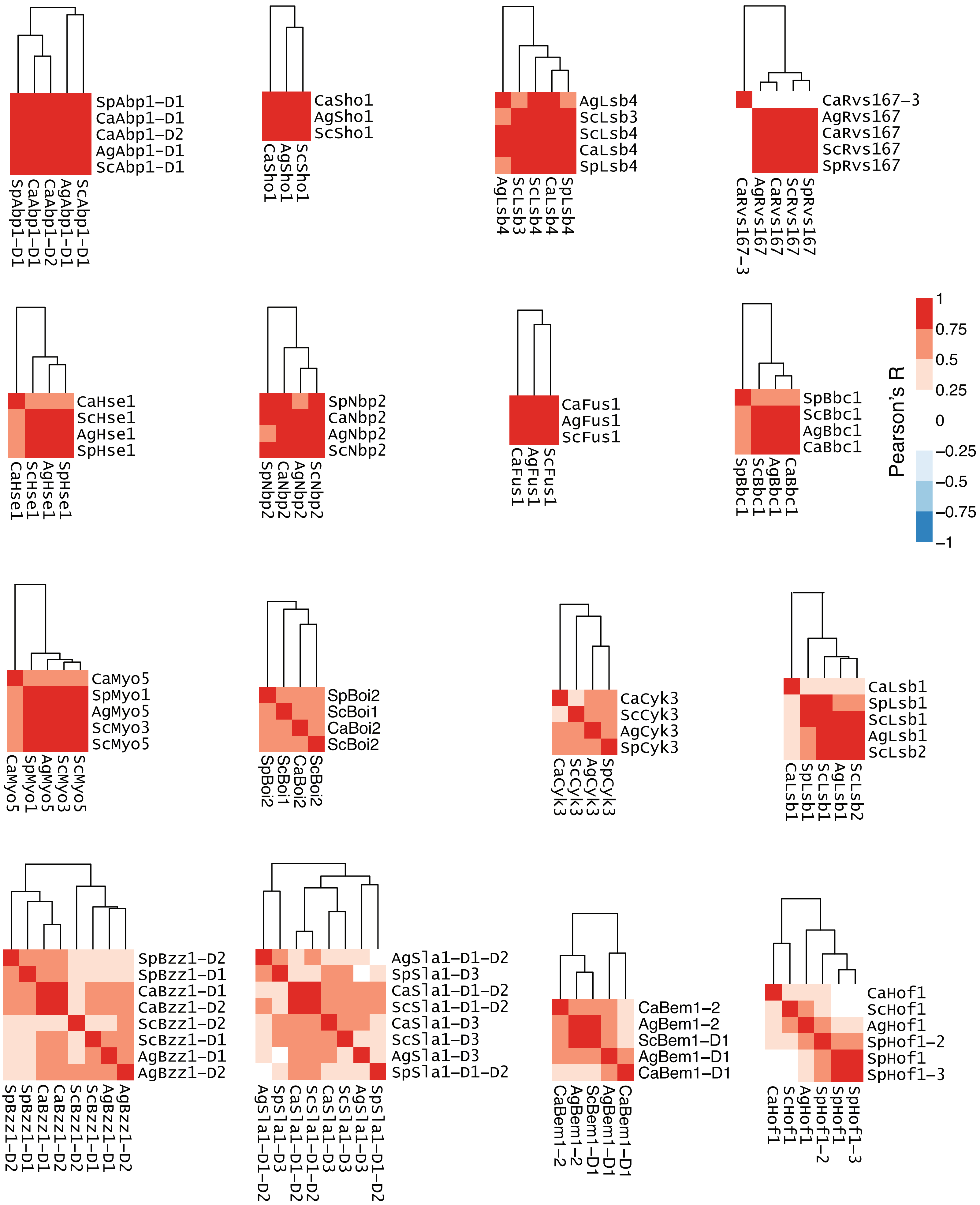
SpAbp1−D1
CaAbp1−D1
CaAbp1−D2
AgAbp1−D1
ScAbp1−D1
CaSho1
AgSho1
ScSho1
AgLsb4
ScLsb3
ScLsb4
CaLsb4
SpLsb4
CaRvs167−3
AgRvs167
CaRvs167
ScRvs167
SpRvs167
CaHse1
ScHse1
AgHse1
SpHse1
SpNbp2
CaNbp2
AgNbp2
ScNbp2
CaFus1
AgFus1
ScFus1
SpBbc1
ScBbc1
AgBbc1
CaBbc1
Pearson's R
1
0.75
0.5
0.25
0
−0.25
−0.5
−0.75
−1
CaMyo5
SpMyo1
AgMyo5
ScMyo3
ScMyo5
SpBoi2
ScBoi1
CaBoi2
ScBoi2
CaCyk3
ScCyk3
AgCyk3
SpCyk3
CaLsb1
SpLsb1
ScLsb1
AgLsb1
ScLsb2
SpBzz1−D2
SpBzz1−D1
CaBzz1−D1
CaBzz1−D2
ScBzz1−D2
ScBzz1−D1
AgBzz1−D1
AgBzz1−D2
AgSla1−D1−D2
SpSla1−D3
CaSla1−D1−D2
ScSla1−D1−D2
CaSla1−D3
ScSla1−D3
AgSla1−D3
SpSla1−D1−D2
CaBem1−2
AgBem1−2
ScBem1−D1
AgBem1−D1
CaBem1−D1
CaHof1
ScHof1
AgHof1
SpHof1−2
SpHof1
SpHof1−3
