# Supplementary material for: Evolution of the SH3 Domain Specificity Landscape in Yeasts
Source: PLoS One. 2015 Jun 11;10(6):e0129229. doi: 10.1371/journal.pone.0129229 (PMC4466140; doi:10.1371/journal.pone.0129229)
Supplement: S1 Table — (DOC) [file pone.0129229.s005.doc]

**S1 Table. Names of the SH3 proteins used**

***S. cerevisiae***

|  | **Name Used** | **Standard**  **Name** | **Systematic**  **Name** | **Swiss-Prot**  **Name** | **RefSeq**  **Name** |
| --- | --- | --- | --- | --- | --- |
| **1** | ScAbp1 | Abp1 | YCR088W | P15891 | NP_010012 |
| **2** | ScBbc1 | Bbc1 | YJL021C | P47068 | NP_012514 |
| **3** | ScBem1 | Bem1 | YBR200W | P29366 | NP_009759 |
| **4** | ScBoi1 | Boi1 | YBL085W | P38041 | NP_009468 |
| **5** | ScBoi2 | Boi2 | YER114C | P39969 | NP_011039 |
| **6** | ScBud14 | Bud14 | YAR014C | P27637 | NP_009408 |
| **7** | ScBzz1 | Bzz1 | YHR114W | P38822 | NP_011982 |
| **8** | ScCdc25 | Cdc25 | YLR310C | P04821 | NP_013413 |
| **9** | ScCyk3 | Cyk3 | YDL117W | Q07533 | NP_010166 |
| **10** | ScFus1 | Fus1 | YCL027W | P11710 | NP_009903 |
| **11** | ScHof1 | Hof1 | YMR032W | Q05080 | NP_013746 |
| **12** | ScHse1 | Hse1 | YHL002W | P38753 | NP_011861 |
| **13** | ScLsb1 | Lsb1 | YGR136W | P53281 | NP_011652 |
| **14** | ScLsb2 | Pin3 | YPR154W | Q06449 | NP_015480 |
| **15** | ScLsb3 | Lsb3 | YFR024C | P43603 | NP_219497 |
| **16** | ScLsb4 | Ysc84 | YHR016C | P32793 | NP_011880 |
| **17** | ScMyo3 | Myo3 | YKL129C | P36006 | NP_012793 |
| **18** | ScMyo5 | Myo5 | YMR109W | Q04439 | NP_013827 |
| **19** | ScNbp2 | Nbp2 | YDR162C | Q12163 | NP_010446 |
| **20** | ScPex13 | Pex13 | YLR191W | P80667 | NP_013292 |
| **21** | ScRvs167 | Rvs167 | YDR388W | P39743 | NP_010676 |
| **22** | ScSdc25 | Sdc25 | YLL016W | P0CF32 | NP_013413 |
| **23** | ScSho1 | Sho1 | YER118C | P40073 | NP_011043 |
| **24** | ScSla1 | Sla1 | YBL007C | P32790 | NP_009546 |

***A. gossypii***

|  | **Name Used** | **Standard**  **Name** | **Systematic**  **Name** | **Swiss-Prot**  **Name** | **RefSeq**  **Name** |
| --- | --- | --- | --- | --- | --- |
| **1** | AgAbp1 |  | AGL237C | Q751E3 | NP_986430 |
| **2** | AgBbc1 |  | AGR306C | Q74Z97 | NP_986972 |
| **3** | AgBem1 |  | AEL241W | Q758K3 | NP_984620 |
| **4** | AgBem1-2 |  | AGL313C | Q751L4 | NP_986354 |
| **5** | AgBoi2 |  | AGL293C | Q751J9 | NP_986374 |
| **6** | AgBud14 |  | AER220c | Q756N4 | NP_985077 |
| **7** | AgBzz1 |  | ACR266W | Q75BK5 | NP_983668 |
| **8** | AgCdc25 |  | ADL038W | Q75AF6 | NP_984058 |
| **9** | AgCdc25-2 |  | AFR630C | Q752E6 | NP_986177 |
| **10** | AgCyk3 |  | ADL288C | Q75B60 | NP_983808 |
| **11** | AgFus1 |  | AFR699C | Q751X6 | NP_986247 |
| **12** | AgHof1 |  | ABR082W | Q75DE5 | NP_983028 |
| **13** | AgHse1 |  | ABR008C | Q75DS3 | NP_982954 |
| **14** | AgLsb1 |  | AFR320W | Q753J2 | NP_985867 |
| **15** | AgLsb4 |  | AEL017W | Q757L7 | NP_984844 |
| **16** | AgMyo5 |  | AEL306C | Q758Q9 | NP_984554 |
| **17** | AgNbp2 |  | AGL169c | Q750V8 | NP_986498 |
| **18** | AgPex13 |  | ACR230C | Q75BP1 | NP_983632 |
| **19** | AgRvs167 |  | AFR140C | Q754D0 | NP_985687 |
| **20** | AgRvs167-2 |  | AER140C | Q756W1 | NP_984999 |
| **21** | AgSho1 |  | AGL286C | Q751J2 | NP_986381 |
| **22** | AgSla1 |  | AGR170c | Q74ZM8 | NP_986836 |

***C. albicans***

|  | **Name Used** | **Standard**  **Name** | **Systematic**  **Name** | **Swiss-Prot**  **Name** | **RefSeq**  **Name** |
| --- | --- | --- | --- | --- | --- |
| **1** | CaAbp1 | CaAbp1 | orf19.2699 | Q5AFA8 | XP_720452 |
| **2** | CaBbc1 | CaBbc1 | orf19.2791 | Q59PV2 | XP_711733 |
| **3** | CaBem1 | CaBem1 | orf19.4645 | Q5AMK1 | XP_722716 |
| **4** | CaBem1-2 |  | orf19.177 | Q59KL0 | XP_710279 |
| **5** | CaBoi2 | CaBoi2 | orf19.3230 | Q5A898 | XP_717921 |
| **6** | CaBud14 | CaBud14 | orf19.3555 | Q59ZG4 | XP_714914 |
| **7** | CaBzz1 | CaBzz1 | orf19.1699 | Q5AJM1 | XP_721771 |
| **8** | CaCdc25 | CaCSC25^1^ | orf19.6926 | P43069 | XP_712727 |
| **9** | CaCdc25-2 | CaBud5 | orf19.1842 | Q59MZ2 | XP_711071 |
| **10** | CaCyk3 |  | orf19.13620 | Q5AAS0 | XP_718745 |
| **11** | CaFus1 | CaFus1 | orf19.1156 | Q59TT8 | XP_713023 |
| **12** | CaHof1 | CaHof1 | orf19.5664 | Q59UQ6 | XP_713345 |
| **13** | CaHse1 | CaHse1 | orf19.3233 | Q5A895 | XP_717924 |
| **14** | CaLsb1 | CaPin3 | orf19.5956 | Q5ANE7 | XP_723167 |
| **15** | CaLsb4 |  | orf19.4127 | **Q59KL5** | XP_710285 |
| **16** | CaMyo5 | CaMyo5 | orf19.738 | Q59MN7 | XP_710986 |
| **17** | CaNbp2 | CaNbp2 | orf19.6588 | Q5AGV0 | XP_721424 |
| **18** | CaPex13 | CaPex13 | orf19.7282 | Q5A425 | XP_716417 |
| **19** | CaRvs167 | CaRvs167 | orf19.1220 | Q59LF3 | XP_710570 |
| **20** | CaRvs167-2 |  | orf19.4742 | Q5AQ66 | XP_723429 |
| **21** | CaRvs167-3 |  | orf19.1861 | Q59U90 | XP_713118 |
| **22** | CaScp1^2^ |  | orf19.6279 | Q5AAN3 | XP_718781 |
| **23** | CaSho1 | CaSsu81 | orf19.4772 | Q5AQ36 | XP_723458 |
| **24** | CaSla1 | CaSla1 | orf19.1474 | Q5ALV2 | XP_722523 |

***S. pombe***

|  | **Name Used** | **Standard**  **Name** | **Systematic**  **Name** | **Swiss-Prot**  **Name** | **RefSeq**  **Name** |
| --- | --- | --- | --- | --- | --- |
| **1** | SpAbp1 | Abp1, App1 | SPAPJ760.02c | Q9P7E8 | NP_594921 |
| **2** | SpBbc1 |  | SPAC23A1.17 | O42854 | NP_594446 |
| **3** | SpBem1 | Scd2, Ral3 | SPAC22H10.07 | P40996 | NP_593744 |
| **4** | SpBoi2 | Pob1 | SPBC1289.04c | O74653 | NP_596828 |
| **5** | SpBud14 | Tea4, Wsh3 | SPBC1706.01 | O60132 | NP_595240 |
| **6** | SpBzz1 | Bzz1 | SPBC12C2.05c | Q09746 | NP_596017 |
| **7** | SpCdc25 | Ste6 | SPCC1442.01 | P26674 | NP_588316 |
| **8** | SpCyk3 | Cyk3 | SPAC9G1.06c | O14302 | NP_593561 |
| **9** | SpHof1 | Imp2 | SPBC11C11.02 | Q10199 | NP_596391 |
| **10** | SpHof1-2 | Cdc15 | SPAC20G8.05c | Q09822 | NP_593322 |
| **11** | SpHof1-3 | Sfp47 | SPAC7D4.02c | O14259 | NP_593857 |
| **12** | SpHse1 | Hse1 | SPBC1734.08 | O74749 | NP_595425 |
| **13** | SpLsb1 | Csh3 | SPBC119.05C | O43125 | NP_595286 |
| **14** | SpLsb4 |  | SPAPJ696.02 | Q9URW6 | NP_593048 |
| **15** | SpMyo1 | Myo1 | SPBC146.13c | Q9Y7Z8 | NP_595402 |
| **16** | SpNbp2 | Skb5 | SPCC24B10.13 | Q9US59 | NP_588016 |
| **17** | SpPex13 | Pex13 | SPAC3C7.10 | O14136 | NP_593611 |
| **18** | SpRvs167 | Hob1 | SPBC21D10.12 | O74352 | NP_596001 |
| **19** | SpScp1^2^ | Mug137 | SPCC1919.11 | O94478 | NP_588493 |
| **20** | SpScp2^2^ |  | SPBC19C2.10 | Q9UUD0 | NP_595695 |
| **21** | SpSla1 | Shd1, Sla1 | SPAC16E8.01 | O13736 | NP_594213 |

*Saccharomyces* Genome Database (http://www.yeastgenome.org), *Ashbya* Genome Database (http://agd.vital-it.ch), *Candida* Genome Database (http://www.candidagenome.org), PomBase (http://www.pombase.org), UniProt (http://www.uniprot.org)

^1^C. albicans Suppressor of Cdc25

^2^SH3 Containing Protein
